# Targeting autophagy as a therapeutic strategy for identification of liganans from *Peristrophe japonica* in Parkinson’s disease

**DOI:** 10.1038/s41392-020-00442-x

**Published:** 2021-02-17

**Authors:** An-Guo Wu, Rong Pan, Betty Yuen-Kwan Law, Wen-Qiao Qiu, Jian-Ming Wu, Chang-Long He, Vincent Kam-Wai Wong, Chong-Lin Yu, Xiao-Gang Zhou, Da-Lian Qin

**Affiliations:** 1 Sichuan Key Medical Laboratory of New Drug Discovery and Druggability Evaluation, Luzhou Key Laboratory of Activity Screening and Druggability Evaluation for Chinese Materia Medica, School of Pharmacy, Department of Human Anatomy, School of Preclinical Medicine, Education Ministry Key Laboratory of Medical Electrophysiology; Southwest Medical University, Luzhou, 646000 China; 2 State Key Laboratory of Quality Research in Chinese Medicine, Macau University of Science and Technology, Macau, China

**Keywords:** Drug discovery, Neuroscience

**Dear Editor,**

PD is characterized by the loss of dopaminergic neurons in substantia nigra, and the loss of dopamine resulting in motor deficit.^1^ Its main pathological hallmarks include the genetic mutations of gene such as α-synuclein.^2^ Increasing study showed that the dopaminergic neurons in midbrain are sensitive and damaged by the PD toxins. 6-Hydroxydopamine (6-OHDA) is widely used to induce lesion of nigrostriatal dopaminergic system in the PD model systems, including nerve cells, *Caenorhabditis elegans* (*C. elegans*), and rats.^3^ Autophagy (mitophagy) is a cellular self-digestive process that engulfs the damaged organelles, including injured mitochondria and misfolded proteins such as α-synuclein.^4^ Therefore, enhanced autophagy plays an important role in neuroprotection via cellular degradation of damaged mitochondria or mutant proteins in neurodegenerative diseases such as PD. *Peristrophe japonica* (PJ), a traditional Chinese medicine, is reported to exhibit anti-inflammatory, anti-bacterial, and anti-oxidative effects.^5^ However, the neuroprotective components of PJ and the mechanism remain un-elucidated.

In this study, we found that the total ethanol extract of PJ (PJ-TEE) could protect against 6-OHDA-induced damage in PC-12 cells (Supplementary Fig. 1). Meanwhile, PJ-TEE exhibited potent autophagy effect by dose-dependently increasing the number of GFP-LC3 puncta formation in stable RFP-GFP-LC3 U87 cells (Supplementary Fig. 2a). Based on the autophagy activity-guided chemical separation, PJ-TEE was extracted using petroleum ether, ethyl acetate, and n-butanol reagents, and the components in these fractions were analyzed by UHPLC-DAD-TOF/MS (Supplementary Fig. 2b). In stable RFP-GFP-LC3 U87 and PC-12 cells, ethyl acetate fraction (EF) was demonstrated to induce the strongest autophagy effect (Supplementary Fig. 2c–e). After sub-fractionation of EF to 34 fractions, F11 to F34 were found to induce autophagy in stable RFP-GFP-LC3 U87 and PC-12 cells (Supplementary Fig. 3). Finally, 3 liganans, including justicidin A (JA), justicidin B (JB), and justicidin C (JC) (Fig. 1a) in F11, were isolated and identified by UHPLC-DAD-TOF/MS and nuclear magnetic resonance (NMR) instruments (Supplementary Fig. 4). To further confirm the autophagy effect of F11 was attributed to JA, JB, and JC, stable RFP-GFP-LC3 U87, PC-12, and SHSY5Y cells were adopted. The results showed that JA, JB, and JC dose-dependently increased the number of GFP-LC3 puncta formation in stable RFP-GFP-LC3 U87 cells (Fig. 1b), and LC3-II protein expression in both PC-12 and SHSY5Y cells (Fig. 1c and Supplementary Fig. 5 and 7). Among them, JA time-dependently increased the ratio of RFP-LC3/GFP-LC3 puncta in stable RFP-GFP-LC3 U87 cells (Supplementary Fig. 6 and Video 1). Meanwhile, JA, JB, and JC increased the ratio of RFP-LC3/GFP-LC3 puncta and the number of autophagosome in PC-12 cells (Supplementary Fig. 8a, b). In addition, LY294002 (LY) and Bafilomycin A1 (Baf) inhibited the autophagic sequestration and autophagosome-lysosome fusion induced by JA, JB, and JC (Fig. 1e and Supplementary Fig. 8c, d). All these evidences suggested that the liganans induced autophagic flux in neurons. Then, the mechanistic study demonstrated that JA, JB, and JC activated autophagy via the AMPK/ULK1, Raf/MEK/ERK, but mTOR-independent signaling pathways (Fig. 1d and Supplementary Fig. 9, 10). In addition, compound C (CC, an AMPK inhibitor) and SCH772984 (SCH, an ERK inhibitor) decreased the number of GFP-LC3 puncta formation in stable RFP-GFP-LC3 U87 cells and LC3-II protein expression in PC-12 cells (Fig. 1e and Supplementary Fig. 11). Furthermore, JA, JB, and JC increased the number of GFP-LC3 puncta formation and LC3-II protein expression in MEF Atg7^+/+^ but not in MEF Atg7^−/−^ cells (Fig. 1f and Supplementary Fig. 12, 13), suggesting that JA, JB, and JC induced autophagy via Atg7. Moreover, Parkin/PINK1-mediated mitophagy was also activated by JA, JB, and JC as revealed by the colocalization of MitoTracker with GFP-LC3 puncta and the decreased GFP/RFP ratio illuminated by mCherry-GFP-FIS1101-152 (mito-QC) (Supplementary Fig. 14, 15 and Video 2).

**Fig. 1:**
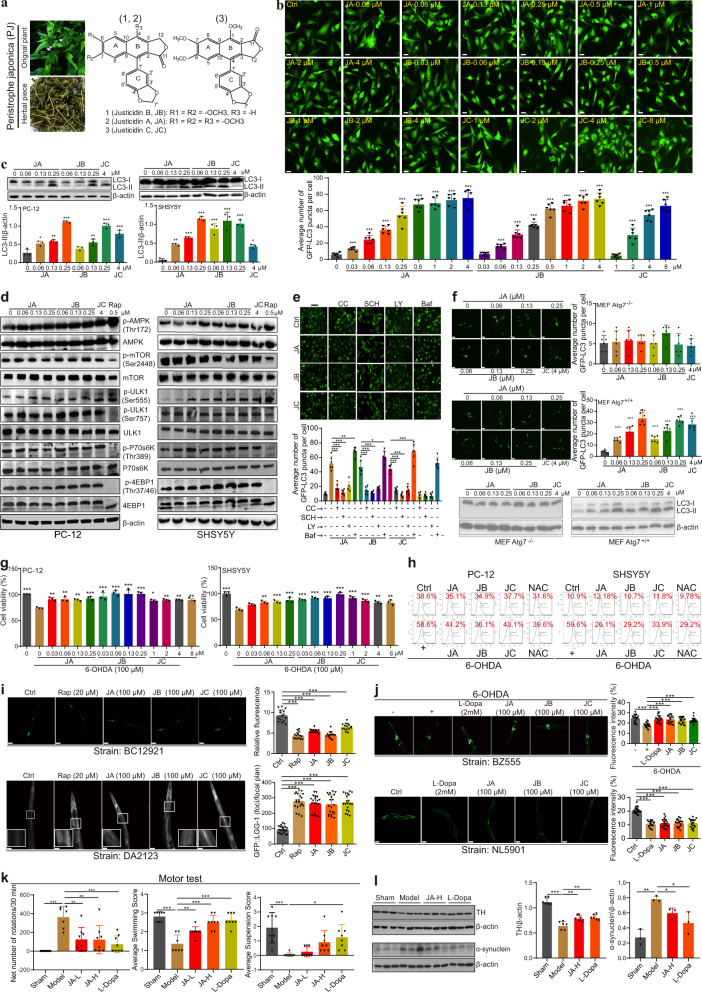
The liganans, including JA, JB, and JC, isolated from PJ exert neuroprotective effect in nerve cell, C. elegans, and rat models of PD. **a** Pictures of the original plant and dried herbal piece of *Peristrophe japonica*, and the chemical structures of JA, JB, and JC. **b** Representative images of cells with GFP or GFP-LC3 puncta, and the average number of GFP-LC3 puncta per cell in JA, JB, or JC treated stable RFP-GFP-LC3 U87 cells at 24 h (scale bar: 50 μm). **c** Protein expression and quantification of LC3-II in JA, JB, or JC treated PC-12 and SHSY5Y cells at 24 h. **d** Protein expression of the phosphorylation and total forms of AMPK, mTOR, ULK1, P70s6K, and 4EBP1 in JA, JB, JC, or Rapamycin (Rap) treated PC-12 and SHSY5Y cells at 24 h. **e** Representative images of cells with GFP or GFP-LC3 puncta, and the average number of GFP-LC3 puncta per cell in stable RFP-GFP-LC3 U87 cells treated with JA (0.13 μM), JB (0.13 μM), or JC (4 μM) in the presence or absence of CC (2.5 μM), SCH (10 μM), LY (25 μM), or Baf (1 nM) for 24 h (scale bar: 50 μm). **f** Upper: representative images of cells with GFP or GFP-LC3 puncta, and the average number of GFP-LC3 puncta per cell in GFP-LC3 transiently transfected MEF Atg7^−/−^ and MEF Atg7^+/+^ cells. Below: protein expression of LC3-II in JA, JB, or JC treated MEF Atg7^−/−^ and MEF Atg7^+/+^ cells at 24 h (scale bar: 50 μm). **g** Cell viability of 6-OHDA-induced PC-12 and SHSY5Y cells treated with JA, JB, or JC for 24 h was measured by MTT assay. **h** ROS production of 6-OHDA-induced PC-12 cells or SHSY5Y cells treated with JA (0.13 μM), JB (0.13 μM), JC (4 μM), or NAC (1 mM) for 24 h was measured by flow cytometer using H_2_DCFDA reagent. **i** Representative images and quantification of p62 and GFP-LGG-1 puncta in BC12921 and DA2123 *C. elegans* strains treated with JA, JB, or JC (scale bar: 200 or 100 μm). **j** GFP expression in dopaminergic neurons of 6-OHDA-induced BZ555 strain and YFP-α-synuclein expression of NL5901 strain treated with L-Dopamine (L-Dopa), JA, JB, or JC (scale bar: 100 μm). **k** Motor performances including net number of rotation in 30 min, average swimming score, and average forelimb hanging score of 6-OHDA rats administrated with L-Dopa or JA. **l** Protein expression and quantification of TH and α-synuclein in brain corpus striatum of 6-OHDA-induced rats treated with L-Dopa or JA. All the experiments were performed in triplicates and data represented the mean ± SEM (*p < 0.05, **p < 0.01, ***p < 0.001)

Next, the neuroprotective effect of JA, JB, and JC was examined in 6-OHDA-induced PC-12 and SHSY5Y cells. Our results showed that JA, JB, and JC dose-dependently increased the cell viability (Fig. 1g), improved cell morphology, and inhibited cell apoptosis (Supplementary Fig. 16a–d). Consistently, LY and CC could attenuate the effect of JA, JB, and JC on the improvement of cell viability (Supplementary Fig. 16e–h). The flow cytometric result demonstrated that JA, JB, and JC significantly decreased ROS production in 6-OHDA-induced PC-12 and SHSY5Y cells (Fig. 1h and Supplementary Fig. 16i, j). In addition, JA, JB, and JC could protect against oxidative damage in H_2_O_2_-induced PC-12 and SHSY5Y cells (Supplementary Fig. 17). Furthermore, JA, JB, and JC inhibited the oligomerization of α-synuclein in PC-12 cells and decreased the levels of WT-, A53T-, A30P-, and E46K-α-synuclein in MEF cells via Atg7 (Supplementary Fig. 18). However, JA, JB, and JC neither inhibited MAO activity nor activated muscarinic (M) receptor (Supplementary Fig. 19). Taken together, JA, JB, and JC exerted neuroprotective effect in 6-OHDA-, H_2_O_2_-, and α-synuclein-induced cell models of PD.

To validate the autophagy and neuroprotective effect of JA, JB, and JC in vivo, both *C. elegans* and rat models of PD were employed. BC12921 strain expressing GFP-p62 fusion protein and DA2123 strain expressing GFP-LGG-1 fusion protein were used to detect the autophagic activity in *C. elegans*. The results showed that JA, JB, and JC significantly decreased the expression of p62 and increased GFP-LGG-1 punctate formation (Fig. 1i). Then, the neuroprotective effect of JA, JB, and JC was evaluated in 6-OHDA-induced BZ555 strain expressing GFP in its dopaminergic neurons, and NL5901 strain expressing human α-synuclein protein tagged with YFP in the body wall muscle. The results showed that JA, JB, and JC significantly decreased the ROS levels (Supplementary Fig. 20a), inhibited the degeneration of dopaminergic neurons (Fig. 1j), and improved the food sensing ability (Supplementary Fig. 20b) in 6-OHDA-induced BZ555 strain, as well as accelerated the degradation of ɑ-synuclein and improved the mobility in NL5901 strain (Fig. 1j, Supplementary Fig. 20c and Video 3). Furthermore, JA was demonstrated to improve dopaminergic neurons in 6-OHDA-induced BZ555, degrade α-synuclein, and improve movement in NL5901 strain via the key autophagy-related genes, including unc-51 and vps-34 (Supplementary Fig. 21). Moreover, motor performance evaluated by apomorphine (APO)-induced asymmetric rotation was tested in 6-OHDA-induced PD rat model (Supplementary Fig. 22). After the administration of JA, the net number of rotation in 30 min was decreased, while the swimming score and the forelimb hanging time were increased (Fig. 1k, Supplementary Video 4–6). At the same time, JA significantly restored TH expression and inhibited α-synuclein expression (Fig. 1l and Supplementary Fig. 23), and activated the Parkin/PINK1 pathway in brain corpus striatum of 6-OHDA-induced rats (Supplementary Fig. 24). Taken together, this study has identified the novel autophagy activators JA, JB, and JC which exert neuroprotective effect in multiple in vivo PD models.

Overall, the liganans, including JA, JB, and JC, were successfully isolated by the autophagy activity-guided purification, and were demonstrated as the main bioactive components in PJ which induced autophagy via Atg7, AMPK/ULK1, Raf/MEK/ERK, but mTOR-independent signaling pathways in PC-12 and SHSY5Y cells. In addition, JA, JB, and JC induced mitophagy via the Parkin/PINK1 pathway. Moreover, JA, JB, and JC exhibited potent neuroprotective effect in nerve cell, *C. elegans*, and rat PD models (Supplementary Fig. 25). Therefore, our study has identified the novel autophagy enhancers from PJ, and demonstrated their neuroprotective effects in vitro and in vivo. These findings suggest that the active liganans in PJ may serve as the potential candidates for PD.

## Supplementary information

Supplementary data

Video S1

Video S2

Video S3

Video S4

Video S5

Video S6

## Data Availability

The data that support the findings of this study are available from the corresponding author upon reasonable request.
