# Supplementary material for: Targeting autophagy as a therapeutic strategy for identification of liganans from Peristrophe japonica in Parkinson’s disease
Source: Signal Transduct Target Ther. 2021 Feb 17;6:67. doi: 10.1038/s41392-020-00442-x (PMC7887264; doi:10.1038/s41392-020-00442-x)
Supplement: Supplementary file 1 — Supplementary data [file 41392_2020_442_MOESM1_ESM.pdf]

Supplementary Materials for

Targeting autophagy as a therapeutic strategy for identification of liganans from *Peristrophe japonica* in Parkinson's disease

An-Guo Wu [1†], Rong Pan [1†], Betty Yuen-Kwan Law [2†], Wen-Qiao Qiu [1], Jian-Ming Wu [1], Chang-Long He [1], Vincent Kam-Wai Wong [2], Chong-Lin Yu [1*], Xiao-Gang Zhou [1*], Da-Lian Qin[1*]

[1] *Sichuan Key Medical Laboratory of New Drug Discovery and Druggability Evaluation, Luzhou Key Laboratory of Activity Screening and Druggability Evaluation for Chinese Materia Medica, School of Pharmacy; Department of Human Anatomy, School of Preclinical Medicine; Education Ministry Key Laboratory of Medical Electrophysiology; Southwest Medical University, Luzhou, 646000, China;*
[2] *State Key Laboratory of Quality Research in Chinese Medicine, Macau University of Science and Technology, Macau, China.*

† These authors contributed equally to this work.
*Correspondence e-mail: 8056ycl@swmu.edu.cn to Chong-Lin Yu
zxg@swmu.edu.cn to Xiao-Gang Zhou
dalianqin@swmu.edu.cn to Da-Lian Qin

This file includes:

Materials and Methods

Supplemental Figures

Supplementary Fig. 1 PJ-TEE protects against 6-OHDA-induced damage in PC-12 cells.
Supplementary Fig. 2 EF isolated from PJ-TEE induces the highest autophagic activity.
Supplementary Fig. 3 F11-F34 isolated from EF of PJ-TEE induce autophagy.
Supplementary Fig. 4 Isolation and elucidation of JA, JB and JC.
Supplementary Fig. 5 Cytotoxicity of JA, JB and JC in PC-12 cells or SHSY5Y cells after 48 h of treatment were determined by MTT assay.
Supplementary Fig. 6 JA increases autophagic flux in stable RFP-GFP-LC3 U87 cells.

Supplementary Fig. 7 Cell viability of JA, JB and JC in PC-12 cells or SHSY5Y cells after 24 h of treatment were determined by a flow cytometer using the Annexin V-FITC/PI Apoptosis detection Kit.
Supplementary Fig. 8 JA, JB and JC induce autophagy flux in PC-12 cells.
Supplementary Fig. 9 The relative protein expression of p-AMPK (Thr172), p-mTOR (Ser2448), p-ULK1 (Ser555), p-ULK1 (Ser757), p-P70s6K (Thr389) or p-4EBP1 (Thr37/46) to β-actin in PC-12 cells and SHSY5Y cells.
Supplementary Fig. 10 JA, JB and JC activate the Raf/MEK/ERK signaling pathway.
Supplementary Fig. 11 Compound C (CC) and SCH772984 (SCH) inhibit JA-induced LC3-II expression in PC-12 cells.
Supplementary Fig. 12 Cytotoxicity of JA, JB and JC in Atg7-knockout MEF cells and wild type MEF cells after 48 h of treatment were determined by MTT assay.
Supplementary Fig. 13 The relative protein expression of LC3-II to β-actin in Atg7-knockout MEF cells and wild type MEF cells.
Supplementary Fig. 14 JA, JB and JC activate mitophagy in PC-12 cells.
Supplementary Fig. 15 JA, JB and JC activate the Parkin/PINK1 signaling pathway.
Supplementary Fig. 16 JA, JB and JC increase the cell viability of 6-OHDA-induced PC-12 and SHSY5Y cells via autophagy induction.
Supplementary Fig. 17 JA, JB and JC rescue PC-12 cells or SHSY5Y cells from cell death induced by H_2O_2.
Supplementary Fig. 18 JA, JB and JC inhibit the oligomerization of α-synuclein in PC-12 cells and decreased the levels of mutant α-synuclein in MEF cells via Atg7.
Supplementary Fig. 19 The effect of JA, JB and JC on the activity of MAO and the activation of M receptor.
Supplementary Fig. 20 JA, JB and JC possess anti-oxidative and behavioral improvement effects in BZ555 and NL5901 strains.
Supplementary Fig. 21 JA exerts neuroprotective effects in BZ555 and NL5901 *C. elegans* via the autophagy-related genes *unc-51* and *vps-34*.
Supplementary Fig. 22 Design of the animal experiment in 6-OHDA-induced rats.
Supplementary Fig. 23 Immunohistochemistry detection of α-synuclein in the corpus striatum of 6-OHDA-induced rats treated with L-Dopa or JA.
Supplementary Fig. 24 JA activates the Parkin/PINK1 signaling pathway in 6-OHDA-induced rats.
Supplementary Fig. 25 A schematic diagram of the study on PJ.
Supplementary Fig. 26 The full-length Western blotting images for **Supplementary Fig. 2e** and **3c**.
Supplementary Fig. 27 The full-length Western blotting images for **Fig. 1c**.
Supplementary Fig. 28 The full-length Western blotting images for **Supplementary Fig. 8b, c**.

Supplementary Fig. 29 The full-length Western blotting images for **Fig. 1d**.
Supplementary Fig. 30 The full-length Western blotting images for **Supplementary Fig. 10**.
Supplementary Fig. 31 The full-length Western blotting images for **Supplementary Fig. 11**
Supplementary Fig. 32 The full-length Western blotting images for **Fig. 1f**.
Supplementary Fig. 33 The full-length Western blotting images for **Supplementary Fig. 15**.
Supplementary Fig. 34 The full-length Western blotting images for **Supplementary Fig. 19**.
Supplementary Fig. 35 The full-length Western blotting images for **Fig. 1l**.
Supplementary Fig. 36 The full-length Western blotting images for **Supplementary Fig. 24**.

Supplementary Videos

Supplementary Video 1. The real-time autophagy flux induced by JA in stable RFP-GFP-LC3 U87 cells for 24 h.
Supplementary Video 2. The real-time mitophagy induced by JA in transiently transfected mito-QC PC-12 cells.
Supplementary Video 3. The real-time mobility of worms of NL5901 strain *C. elegans* treated with JA.
Supplementary Video 4. The real-time rotation of rats in sham, 6-OHDA, JA-L, JA-H, or L-Dopa group.
Supplementary Video 5. The real-time swimming of rats in the sham, 6-OHDA, JA-L, JA-H, or L-Dopa group.
Supplementary Video 6. The real-time forelimb hanging of rats in the sham, 6-OHDA, JA-L, JA-H, or L-Dopa group.

Materials and Methods

Reagents, plasmids and antibodies

Compound C (CC, T1977), LY294002 (LY, T2008), SBI-0206965 (SBI, T2128), atropine (Ato, T0375), and selegiline hydrochloride (T5050) were purchased from Topscience Company., Ltd. (Shanghai, China). Bafilomycin A1 (Baf, HY-100558) was purchased from MCE chemical (Shanghai, China). H_2DCFDA fluorescence probe was bought from Invitrogen (Carlsbad, CA, USA). Annexin V-FITC/PI Apoptosis detection Kit was bought from 4A Biotech Co., Ltd. (Beijing, China). Mito-Tracker Red CMXRos was purchased from Beyotime Institute of Biotechnology (Shanghai, China). 3-(4,5-dimethylthiazol-2-yl)-2,5-dimethyltetrazolium bromide (MTT, M2128) and Crystal violet (C6158) were obtained from Sigma-Aldrich (St. Louis, MO, USA). Mouse monoamine oxidase (MAO) ELISA kit (JL20303) and mouse muscarinic acetylcholine receptor (M-AChR) ELISA kit (JL20322) were purchased from Jianglai Biological Technology Co., Ltd. (Shanghai, China). Methanol and acetonitrile were purchased from Adamas-beta® Reagent, Ltd. Milli-Q water was prepared by the Milli-Q integral water purification system (Millipore, Billerica, MA, USA). pEGFP-LC3 and mRFP-GFP Tandem Fluorescent-Tagged LC3 (tfLC3) plasmids were generous gifts obtained from Prof. Tamotsu Yoshimori (Osaka University, Japan). Antibodies against p-AMPK (Thr172, #2535), AMPK (#2532), p-mTOR (Ser2448, #5536), mTOR (#2983), p-ULK1 (Ser757, #6888), p-ULK1 (Ser555, #5869), p-P70s6K (Thr389, #9205), P70s6K (#9202), p-B-Raf (Ser445, #2696), B-Raf (#14814), p-MEK1/2 (Ser217/221, #9154), MEK1/2 (#4694), p-Erk1/2 (Thr202/Tyr04, #4370) and Erk1/2 (#4695) were purchased from Cell Signaling Technology Inc, (CST, Beverly, MA, USA). p-4EBP1 (Thr37/46, AP0030) and 4EBP1 (A1248) were bought from ABclonal Biotechnology Co., Ltd. (Wuhan, China). LC3 (PM036) antibody was purchased from MBL International, (MA, USA). ULK1 (sc-390904), Parkin (sc-32282) and β-actin (sc-47778) antibody was bought from Santa Cruz Biotechnology (Texas, USA). PINK1 (23274-1-AP) antibody was purchased from Proteintech Group, Inc., (Wuhan, China).

Plant material

The herb of PJ was bought from Yongyi Traditional Chinese Medicine Co., Ltd. (Hebei, China). A voucher specimen (No. MUST-TR201508) was deposited at the State Key Laboratory of Quality Research in Chinese Medicine, Macau University of Science and Technology.

Extraction and isolation

2.5 kg of Air-dried PJ was cut into pieces and extracted by 10 times its volume of 75% ethanol for 1.5 h using the refluxing method, which was repeated for 3 times. After extraction, all the solutions were then combined, filtered, and concentrated by a rotary evaporator under vacuum condition at 60 °C. The obtained crude extract was re-dissolved in water and partitioned with petroleum ether (1:1 vol/vol) for 3 times, then collected the petroleum ether layer. The remained water fraction was then partitioned with ethyl acetate (1:1 vol/vol) for 3 times to obtain ethyl acetate layer. Finally, the remained water fraction was partitioned once again with n-butanol (1:1 vol/vol) for 3 times to get the n-butanol layer. All the solvents were removed and dried under the vacuum. Then the fractions including petroleum ether fraction (PF, 136g), ethyl acetate (EF, 203 g), n-butanol fraction (NF, 108 g), and water fraction (WF, 144 g) were obtained. Further isolation of EF was performed by using column chromatography filled with silica gel (40-60 μm, Grace, USA), which was paralleled with the detection by thin layer chromatography (TLC) using F254 plates pre-coated with 60 μm silica gel, then 34 fractions were collected. Among them, the compounds in F11 (322 mg) were separated on a C_{18} column (Thermo fisher, 250 mm × 10 mm, particle size: 5 μm, flow rate: 3.0 mL/min), and analyzed by the preparative high-performance liquid chromatography (Pre-HPLC) equipped with a 1200 quaternary pump and a 1200 diode-array detector (Agilent, USA) with the mobile phase was CH_3OH-H_2O (48:52). Finally, three main compounds including compound 1 (32.1 mg), compound 2 (38.2 mg), and compound 3 (10.5) with the purity of ≥98% were obtained.

Chromatography conditions

Analysis of the samples, such as WF, NF, EF, and PF isolated from PJ-TEE, and the further isolated fractions from EF and the three compounds derived from F11 were

performed by 1290 ultra-high performance liquid chromatography (UHPLC) equipped with the Agilent 6230 electrospray ionization (ESI) time-of-flight (TOF) mass spectrometer equipped with a DAD detector (Agilent Technologies, USA) in positive ion mode. The separation was conducted on an Agilent Zorbax Eclipse Plus C_{18} column (particle size: 1.8 μm, flow rate: 0.35 mL/min). The mobile phase consisted of A (0.1% formic acid in water) and B (0.1% formic acid in acetonitrile) with the elution program as follow: 0–7 min, 5–60% B; 7–11 min, 60–100% B; 11–12 min, 100% B; 12–15 min, 5% B. After analysis by the UHPLC-DAD-TOF/MS in the scan mode (m/z 100 to 1700 Da with 2.0 spectra/s), all the data were analyzed using the Agilent MassHunter Workstation software B.01.03.

Identification

Ultraviolet (UV) and HRMS spectrums of JA, JB and JC were obtained by UHPLC-DAD-TOF/MS. Nuclear magnetic resonance (NMR) spectrums including ^{1}H NMR and ^{13}C NMR of JA, JB and JC were recorded by the Bruker Ascend 600 NMR spectrometer at 600 MHz with TMS as an internal standard. The chemical shifts and the coupling constants (J) are expressed in δ (ppm) and hertz (Hz), respectively.

Cell culture

Stable RFP-GFP-LC3 U87 cells were generous gift kindly provided by Dr. Xiaoming Zhu (Macau University of Science and Technology, Macao, China). Wild type Atg7 and Atg7-deficient mouse embryonic fibroblasts (MEF) were kindly provided by Masaaki Komatsu (Juntendo University, School of Medicine, Tokyo, Japan). PC-12 cells and SHSY5Y cells were purchased from the American Type Culture Collection (ATCC) (Rockville, MD, USA). Except the medium of PC-12 cells is DMEM supplemented with 10% horse serum (HS), 5% fetal bovine serum (FBS) and 50 U/mL penicillin and 50 μg/mL streptomycin (Invitrogen, Scotland, UK). Other cells were cultured in the complete medium containing α-MEM or DMEM, 10% FBS, and 50 U/mL penicillin and 50 μg/mL streptomycin. All cells were maintained in the 5% CO_2 incubator with 75% humidity at 37 °C.

Cytotoxicity assays

The test drugs used in this study were dissolved with DMSO and stored at -20 °C until further use. The viability of the cells treated as the indicated drugs was measured by MTT assay using the 3-[4,5-dimethylthiazol-2-yl]-2,5-diphenyl tetrazolium bromide (Sigma-Aldrich, MO, USA) reagent as previously described. Briefly, cells seeded in 96-well microplates were treated the drugs as the indicated concentrations for 24-48 h [1]. After treatments, the medium was removed and 100 μL of fresh medium with 10 μL of MTT solution (5 mg/mL) was added into each well, followed by a further incubation for 4 h. The formed formazan representing the viability of cells was then dissolved by 100 μL DMSO. The solution was then subjected to the spectrophotometer and the colorimetric reading was determined at OD 570 nm. Apart from MTT assay, the viability of cells was also measured by crystal violet staining [2]. In brief, cells seeded in 6-well plate were treated the drugs as the indicated concentrations for 24 h. After treatment, the medium was removed, then the cells were incubated with crystal violet solution for 20 min. The excessive crystal violet solution adhered on the cells was wash away by PBS and air-dried at room temperature. The representing images of stained cells were captured by CCD digital camera Spot RT3™ under the Nikon ECLIPSE 80i microscope. While the cell viability was quantified by dissolving the crystal violet in 10% acetic acid, and the colorimetric reading of the solution was determined by the spectrophotometer at OD 570 nm. The percentage of cell viability by these two methods was calculated using the following formula: Cell viability (%) = $\text{Cells number}_{\text{treated}}/\text{Cells number}_{\text{DMSO control}} \times 100$. Data were obtained from three independent experiments.

Quantification of the number of GFP-LC3 puncta

Quantification of the number of GFP-LC3 puncta in GFP-LC3 transiently transfected MEF cells or stable RFP-GFP-LC3 U87 cells were conducted as previously described [3]. In brief, stable RFP-GFP-LC3 U87 cells or MEF cells were seeded on coverslips in 6-well plates. MEF cells were transfected with pEGFP-LC3 by the Exfect® Transfection Reagent (Vazyme Biotech Co., Ltd., Nanjing, China) according to the manufacture's instruction. Cells were then treated the drugs as the indicated

concentrations for 24 h. After treatment, the cells were fixed by 4% paraformaldehyde (PFA) for 20 min at room temperature, followed by the wash with PBS for 2 times. The slides were then taken out for air-drying and mounted by FluorSave™ mounting media (Calbiochem, San Diego, CA, USA). Representative images of cells were captured by Nikon ECLIPSE 80i fluorescence microscope equipped with a CCD digital camera Spot RT3™ (Diagnostic Instruments, Inc., Melville, NY, USA). The number of GFP-LC3 puncta per cells using ImageJ software. Briefly, the green channel of RGB images was extracted and converted to the grayscale images by auto-thresholding, and GFP-LC3 granules were analyzed for the number. The criteria for the analysis are as follow: all particle sizes larger than background pixelation but smaller than the average nuclear were selected for the analysis, while the cells containing apoptotic nuclei or necrotic cell bodies were excluded from the analysis.

Determination of Autophagy Flux in Stable RFP-GFP-LC3 U87 Cells

The autophagy flux was determined as described previously by calculating the RFP/GFP ratio in stable RFP-GFP-LC3 cells [2]. In brief, stable RFP-GFP-LC3 U87 cells seeded in 6-well plates were treated with JA (0.13 μM). Then, the dynamic change of GFP-LC3 and RFP-LC3 in stable RFP-GFP-LC3 cells was monitored by real-time observation using ImageXpress Micro 4 Widefield High-Content Imaging System (Molecular Devices, San Jose, CA, USA). Finally, a 11 s video that records the autophagy flux induced by JA in stable RFP-GFP-LC3 U87 cells A549 in 24 h was obtained. At the same time, the representative GFP and RFP fluorescence images of RFP-GFP-LC3 U87 cells at the time points of 0, 2, 4, 6, 8, 10, 12, 14, 16, 18, 20, and 22 h were captured. The autophagy flux induced by JA was determined by calculating the GFP/RFP fluorescence ratio using ImageJ software (ImageJ 1.46r; National Institutes of Health, Bethesda, MD, USA) [4].

mRFP-GFP Tandem Fluorescent-Tagged LC3 (tfLC3) Detection

The autophagosome was determined as described previously in transfected tfLC3 plasmid PC-12 cells [5]. In brief, tfLC3 transiently transfected PC-12 cells were seeded on coverslips in 6-well plates. After treatments with JA, JB, JC or rapamycin at the

indicated concentrations for 24 h, cells were fixed with 4% PFA for 20 min at room temperature and mounted by FluorSave™ mounting media for the examination of GFP/RFP-LC3 in cells by a Leica SP8 confocal microscope with a LAS X 3D Visualization (Leica Microsystems Inc., Wetzlar, Germany). The images of cells with GFP-LC3 and RFP-LC3 at the same field were merged to determine the mean number of LC3B puncta per cell and the percentage of autophagosome per cell using ImageJ software (ImageJ 1.46r; National Institutes of Health, Bethesda, MD, USA).

Transmission electron microscopy

JA-, JB-, or JC-induced autophagosome morphology was examined by transmission electron microscopy according to the reported method [2]. Briefly, PC-12 cells were seeded in 100 mm dishes and treated with JA (0.13 μM), JB (0.13 μM), JC (4 μM), or rapamycin (0.5 μM) for 24 h. After treatment, PC-12 cells were collected and centrifugation at 1000 r/min for 5 min. The supernatant was discarded and the cells were resuspended by 0.5% glutaraldehyde fixative solution and stood still for 10 min at 4 °C. Then, cell suspension was centrifugated at a high speed of 12000 r/min for 10 min. The cell pellet was fixed by adding 3% glutaraldehyde fixative overnight, followed by a wash with PBS. Then, the cells were subjected to a post-fixation with 1% OsO4 and an embedment using Araldite 502. The obtained ultrathin sections were double stained with 3% uranyl acetate and lead citrate, then analyzed by a transmission electron microscope (JEM-1400plus, JEOL, Tokyo, Japan) at a voltage of 80 kV.

Colocalization of GFP-LC3 and Mito-Tracker Red CMXRos

The mitophagy was determined by visualizing the colocalization of GFP-LC3 and Mito-Tracker Red CMXRos in PC-12 cells [6]. In brief, PC-12 cells seeded on 6-well plates were transiently transfected with GFP-LC3 plasmid for 24 h, and followed by the treatment of JA, JB, JC or AICAR for 24 h. Then, cells were incubated with 200 nM Mito-Tracker Red CMXRos solution for 30 min at 37 °C. After incubation, the Mito-Tracker Red CMXRos solution was exchanged by the fresh cell culture medium. The colocalization of GFP-LC3 and Mito-Tracker-Red was visualized and captured using the fluorescence microscope with 100× magnification. The overlap of green and

red dot represents the mitochondrial-containing autolysosome.

Detection of mitophagy using mito-QC plasmid

Mito-QC is made up of a tandem-fusion protein (mCherry-GFP) that is targeted to the outer mitochondrial membrane (OMM) via the mitochondrial targeting sequence of the OMM protein, FIS1. Mito-QC is a pH-sensitive mitochondrial fluorescent probe. The GFP will be quenched when mitophagy is induced because mitochondria are delivered to the lysosome [7]. In this study, we also monitor the mitophagy using mito-QC plasmid. In brief, PC-12 cells seeded on 6-well plates were transiently transfected with mito-QC plasmid for 24 h, and followed by the treatment of JA, JB, JC or AICAR for 24 h. Then, cells were examined and captured by a fluorescence microscope with 10× magnification. The images of cells with GFP and RFP at the same field were merged to determine the mitophagy by calculating the GFP/RFP fluorescence ratio using ImageJ software (ImageJ 1.46r; National Institutes of Health, Bethesda, MD, USA). The intensities of GFP and RFP signals were obtained from 3 randomly selected fields.

Western blot

After drug treatments, cells were lysed by 1× RIPA buffer from Cell Signaling Technologies Inc. (Beverly, MA, USA). The proteins were harvested and the protein concentrations were measured by 1× Bradford reagent (Bio-Rad, Hercules, CA, USA). Equal amount of lysate of each sample was resolved by SDS-PAGE for electrophoresis, then the proteins on the SDS-PAGE gel were transferred to the polyvinylidene difluoride (PVDF) membrane. After that, the membrane was blocked with 5% non-fat milk for 1 h at room temperature, then was incubated with primary antibodies overnight at 4 °C. After incubation, the membrane was washed using TBST for 3 times and further incubated with the HRP-conjugated secondary antibodies for 1 h at room temperature. Then, the membrane was washed using TBST for 3 times, and the band of the targeted proteins was revealed using the ECL Western Blotting Detection Reagents (4A Biotech Co., Ltd, Beijing, China). In this study, the relative quantitation of target protein expression was carried out in the WB images of unsaturated blots and obtained by calculating the normalized band intensities to β-actin using the ImageJ software. All

the data were from for three independent experiments.

Flow cytometric analysis

Measurement of ROS levels

In this study, ROS level in PC-12 or SHSY5Y cells was determined by using flow cytometer as our previously described method [2]. In brief, cells seeded in 6-well plates were treated the indicated drug for 24 h. After treatments, the cells were collected and subjected to centrifugation at 2000 rpm for 5 min. The cells pellet was retained and re-suspended by the fresh medium with 5 μM H_2DCFDA at 37 °C for 30 min. After incubation, the solution was centrifuged at 2000 rpm for 5 min and the cell pellet were washed with PBS. The cells were then analyzed by a FACSVerse flow cytometer (BD Biosciences, San Jose, CA, USA) and the percentage of GFP signal was calculated using the Flowjo software (BD Biosciences, San Jose, CA, USA). Data were obtained from three independent experiments.

Measurement of cell apoptosis

In this study, cell viability and apoptosis of PC-12 or SHSY5Y cells were measured by flow cytometry using the Annexin V-FITC/PI Apoptosis detection Kit from 4A Biotech Co., Ltd. (Beijing, China) [8]. In brief, PC-12 cells seeded in 6-well plates were treated with the indicated drugs for 24 h. After treatments, cells were collected to centrifugate at 2000 rpm for 5 min. Then, the cell pellet was resuspended by the 1× annexin V solution containing FITC and PI reagents, followed an incubation in dark for 15 min. After that, the cells were subjected to the flow cytometric analysis by a FACSVerse flow cytometer according to the manufacturer's instructions. The rate of cell apoptosis or viability was calculated using the Flowjo software. Data were obtained from three independent experiments.

Spectrophotometric monoamine oxidase activity assay

The spectrophotometric monoamine oxidase (MAO) activity assay was measured based on the conversion of benzylamine to benzaldehyde in the role of MAO. Benzaldehyde is generated into aldehydephenylhydrazone which is measured by a spectrophotometer at OD 470 nm. After treatment of JA, JB, JC, or selegiline, PC-12 cells were trypsinzed,

collected and washed by isotonic PBS for 1-2 times, followed by a homogenization with an ultrasonic disintegrator in an ice bath. After centrifugation at a speed of 1000 rpm for 10 min, the supernatant was collected for the determination of the protein concentration using BCA protein concentration determination kit. Then, the MAO activity of the samples was detected by the MAO detection kit (R22030-100T, Shanghai yuanye Bio-Technology Co., Ltd., Shanghai, China) according to the manufacturer's instructions. The MAO activity was calculated using the following formula: MAO activity (U/mg) = benzaldehyde (nmol) × Vt × N/(t×Vs×C). Vt, N, Vs, t, and C represents the total volume (mL) of samples to be detected, the dilution factor (N) of samples, the volume (mL) of samples being detected, the reaction time (min), and the protein concentration of samples, respectively.

Determination of the activation of muscarinic receptor

The effect of JA, one of the liganans, on the activation of muscarinic (M) receptor was performed in ex vivo rabbit intestinal muscle according to reported literature [9]. In brief, after 24 h of fasting, a male New Zealand rabbit was humanely euthanized by means of a blow to the head, 1.5-2 cm of jejunum was removed, washed, freed from mesenteric attachment. The segment of jejunum suspended in the direction of longitudinal and circular smooth muscle fibers in a thermostatically controlled (37 °C) organ bath (10 mL capacity) containing Krebs solution and was continuously gassed with air. The segment of jejunum was connected to an isometric force transducer. Then, the signal output of the mechanical activity was amplified and recorded by the RM6240 series Multi-channel physiological signal acquisition and analysis system (Chengdu Taimeng Technology Co., Ltd. Chengdu, China). Prior to each testing, the segment of jejunum was allowed to equilibrate in Krebs solution. At first, the activation and antagonism of M receptor was validated by 0.01% acetylcholine (Ach) chloride and 0.1% atropine (Atr) sulfate. After wash and equilibrium, 10 mg/mL of JA was added into the Krebs solution, which was followed by the addition of 0.2 mL of 0.01% acetylcholine (Ach) chloride. The signal was recorded by the RM6240 series Multi-channel physiological signal acquisition and analysis system.

C. elegans strains and maintenance conditions

Strains including DA2123 adIs 2122[lgg-1p::GFP::lgg-1+rol-6(su1006)], BC12921 sIs10729 [rCes T12G3.1::GFP + pCeh361], BZ555 egIs1 [dat-1p::GFP], NL5901 pkIs2386 [unc-54p::α-synuclein::YFP + unc-119(+)] were used in this study. All strains were obtained from Caenorhabditis Genetics Center (CGC), and maintained on NGM plates and feed with *Escherichia coli* OP50 at a 20 °C incubator unless otherwise noted.

Determination of autophagy in *C. elegans*

In this study, BC12921 expressing the SQST-1-GFP fusion protein, *C. elegans* homolog of mammal p62 and DA2123 expressing the *C. elegans* microtubule-associated protein 1 light chain 3 beta (LC3B) autophagosome protein LGG-1 fused with GFP were used to detect the autophagic activity in *vivo*. In brief, the strains were treated with JA, JB, JC or Rap as the indicated concentrations for 72 h at 20 °C. After treatment, the animals were washed three times with M9 buffer, and then mounted onto a glass slide to perform microscopic observation and analysis under a fluorescent microscope (Leica DM6B, Leica Microsystems GmbH, Germany). The quantification of p62 expression in BC12921 strain was conducted by measuring the fluorescence intensity representing p62 in the body of worms using ImageJ software. Similarly, the total number of GFP::LGG-1/Atg8 punctae in the same size area was counted in DA2123 strain as previously described [10].

Analysis of intracellular ROS levels in 6-OHDA-treated BZ555 strain

C. elegans BZ555 strain was treated with 6-OHDA (Sigma, St. Louis, MI) in order to induce ROS production [11]. In brief, the synchronized L3 larvae were incubated in solution containing OP50/S-medium mixed with 50 mM 6-OHDA and 10 mM ascorbic acid for 1 h at 22 °C. The solution was mixed gently every 10 min. After 1 h treatment, the worms were washed three times with M9 buffer and then transferred to OP50/NGM plates containing JA, JB, JC, or NAC. At the same time, 0.04 mg/mL 5-fluoro-29-deoxyuridine (FUDR, Sigma, St. Louis, MI) was added into plates to reduce the production of progeny. After 72 h of treatment, the worms were incubated with 2 mM of H_2DCF-DA for 30 min at 20 °C in the dark. Then the worms were collected for the

centrifugation at a speed of 2000 rpm and mounted onto a glass slide in M9 medium with 10 mM sodium azide. The representative images were captured using a fluorescent microscope (Leica DM6B, Leica Microsystems GmbH, Germany), and the ROS levels was measured by quantifying the GFP fluorescence intensity in the worms by ImageJ software. Data were pooled from 20 worms in each group.

Quantitative assay of dopaminergic neurodegeneration

C. elegans BZ555 strain expressing GFP in its dopaminergic neurons was treated with 6-OHDA to induce selective degeneration of dopaminergic neurons [12]. In brief, the synchronized L3 larvae were incubated in solution containing OP50/S-medium mixed with 50 mM 6-OHDA and 10 mM ascorbic acid for 1 h at 22 °C. The solution was mixed gently every 10 min. After 1 h of treatment, the worms were washed three times with M9 buffer and then transferred to OP50/NGM plates containing JA, JB, JC, or levodopa (L-Dopa). At the same time, 0.04 mg/mL FUDR, Sigma, St. Louis, MI was added into plates to reduce the production of progeny. After treatment, the animals were washed three times with M9 buffer and mounted onto a glass slide using 10 mM sodium azide. The representative fluorescence and white images of the neurons in the worms at the same fields were captured and merged under a fluorescence microscope (Leica DM6B, Leica Microsystems GmbH, Germany), and the intensity of fluorescence representing the live dopaminergic neurons was quantified by ImageJ software.

Analysis of food-sensing behavior

The function of *C. elegans* dopaminergic neurons was also assayed by evaluating the food-sensing behavior [13]. In brief, the L3 larvae of BZ555 strain were treated with 6-OHDA or co-treated with 6-OHDA and JA, JB, JC or L-Dopa for 72 h at 20 °C. The worms were then released to the center of NGM agar plate spotted with or without *E. coli* OP50 lawn. After 5 min, the body bends of each worm were counted for 20 s in the presence or absence of food. The slowing rate was calculated according to the following formula:

$$\text{Slowing rate} = (N_{\text{without food}} - N_{\text{with food}})/N_{\text{without food}}$$

where, N represents total number of body bends in the presence or absence of bacterial.

Analysis of movement in *C. elegans*

The motor impairment and dysfunction are the main symptom of PD patients. The strain NL5901 [Punc-54::α-synuclein::YFP; unc-119(+)] expressing human α-synuclein protein tagged with YFP in the body wall muscle displays the progressive decline in body movement [14]. In this study, the improvement of JA, JB and JC in the motor function was evaluated in NL5901 strain. Briefly, age-synchronized NL5901 animals were grown onto NGM plates containing JA, JB, JC or levodopa to reach L4 developmental stage. At the same time, FUDR (50 g/ml) was added to NGM plates to prevent the growth of progeny. When animals grew to L4+5 days, they were placed independently in a drop of M9 buffer, and allowed to recover for 120 s to avoid observing behavior associated with stress. Then the number of body bends was counted in 20 s. Meantime, the representative video of swimming worms in 20 s was captured under a Leica M205FA stereomicroscope (Leica, Wetzlar, Germany) equipped the Bandicam software (Bandicam Company, Seoul, Korea). 20 animals were used to analyze in each experiment, and all experiments were carried out in triplicate.

Quantitative assay of α-synuclein protein aggregation in *C. elegans*

As is known to us, the accumulation of α-synuclein aggregates results in the degeneration and loss of dopaminergic neurons. In this study, the analysis of α-synuclein accumulation were performed in the NL5901 strain [14]. In brief, synchronized L1 larvae were transferred onto the NGM plates containing JA, JB, JC or L-Dopa and incubated for 5 days at 20 °C. Then the animals were collected and the representative images were captured for the analysis of α-synuclein aggregation under the fluorescence microscope. The expression of α-synuclein was measured by quantifying the fluorescence intensity using ImageJ software.

RNAi treatments in *C. elegans*

To examine whether the neuroprotective effect of liganans is associated with the autophagy induction *in vivo*. 6-OHDA-induced BZ555 or NL5901 strain were respectively transferred onto NGM plates which were seeded with the control bacteria HT115 or the RNAi bacteria expressing double-stranded RNA of two key autophagy-

related genes including unc-51 and vps-34. At the same time, L-Dopa (2 mM) or JA (100 μM) was also added into the NGM plates with bacteria HT115 or RNAi bacteria. After treatment, the fluorescence intensity indicating the live dopaminergic neurons in BZ555 worms, and the fluorescence intensity representing the α-synuclein protein aggregation and the movement of NL5901 worms were examined as described above.

6-OHDA-induced motor deficit rat model

Sixty 8-week-old male Sprague-Dawley rats (body weight: 230–250 g) were purchased from the Laboratory Animal Center of Southwest Medical University (Luzhou, China). The animals were housed at 23±2°C with a 40±10% humidity under an automatic 12 h light/dark cycle throughout the acclimatization and experimental periods and given ad libitum access to food and water. All of the animal cares and experiments were reviewed and approved by the Animal Use and Ethics Committee of Southwest Medical University (Luzhou, China). All efforts were made to minimize the animal suffering. 6-OHDA was dissolved in normal saline solution with 0.02% vitamin C to a concentration of 2 μg/μL. After anesthesia, the rats were fixed on a stereotaxic apparatus (DW-2000, Taimeng, Chengdou). Fur on the head was shaved by a pet clipper and 10% povidone-iodine solution was used to sterilize the incision site. The skin was slit and bluntly separated with tissue forceps, and the periosteum was burned with hydrogen peroxide to expose the bregma. According to the Rat Brain Atlas in Stereotaxic Coordinates Paxinos, the exposed skulls were trepanned by a dental drill (3 mm) at the appropriate position. 5 μL of 6-OHDA solution or saline was injected into the right midbrain substantia nigra pars compacta (SNc) at the following stereotaxic coordinates: antero-posterior (AP): −5.0 mm from bregma; medio-lateral (ML): ± 2.0 mm from the midline; dorso-ventral (DV): 7.3 mm from skull using a TJ-2A Syringe Pump Controller. Syringe was lowered into the brain at a rate of 1 mm/min, then the syringe was left in the place for 5 min and the reagents were injected at a rate of 0.2 μL/min. Afterward, the syringe was left in the place for 5 min again before drawing back at a rate of 1 mm/min. The incision was cleaned by povidone-iodine solution and closed using three sutures. 10 IU of penicillin solution was injected intraperitoneally to rats for 1 week so

as to prevent infections. The postoperative behavioral changes of rats including bradykinesia, less movement, tremor, vertical hair, and olfactory abnormality were observed daily. On the 7th, 14th, and 21st day after surgery, apomorphine was subcutaneously injected to induce rotational behavior, and the rats with more than 210 rotations per 30 min or 7 rotations per 1 min in 0.5 h were selected for the following behavioral training and tests.

Groups and administrations

All the rats were randomly divided into 5 groups (n=8 rats/group), including sham, model, 5 mg/kg JA, 10 mg/kg JA, and 10 mg/kg L-Dopa groups. In brief, rats in the sham group were intraperitoneally injected with equal volume of 0.9% normal saline. 6-OHDA-induced rats were intraperitoneally injected with equal volume of 0.9% normal saline, 5 mg/kg JA, 10 mg/kg JA, and L-Dopa. Before 1week of test, all rats were subjected to the following behavioral trainings and tests. All behavioral tests were performed in a double-blinded manner between 10 a.m. and 3 p.m.

Apomorphine-induced rotational test

The apomorphine-induced rotational test was performed as previously described [15]. Briefly, after a subcutaneous injection of apomorphine hydrochloride dissolved in normal saline (0.5 mg/kg; GLPBIO, USA), the full 360-degree rotations of rats within 1 h were manually counted in a cylindrical container (75 cm long, 50 cm wide and 35 cm high). Finally, the net number of rotations was calculated as the positive scores (contralateral rotations) minus the negative scores (ipsilateral rotations).

Swimming test

The swimming test was used to evaluate the limb coordination ability and motility [16]. In brief, rats were placed in a cylindrical swimming pool with a size of 100 cm × 100 cm × 70 cm. The pool was filled with water for 50 cm of depth and the temperature was maintained at 22-25 °C. Swimming test was performed and the score was recorded as previously described [17]. The scoring criteria were as follows: 3 points: continuous swimming, 2.5 points: swimming for most of the time and floating occasionally, 2 points: floating for more than 50% of time, 1.5 points: floating most of the time, 1 point: floating with a few movements of limbs, 0 point: floating without movement of limbs.

The test time of each rat was 10 min, and the time interval of score was 1 min. Three training were performed for each mouse before the test.

Forelimb hanging test

The Forelimb hanging test was used to evaluate the muscular strength and motor neuron integrity [18]. In brief, rats utilized their forelimbs to grab the wire with a diameter of 3 mm and suspend their body on it. The wire was stretched for 30 cm between two posts and height for 70 cm above a soft pad placed on the ground to prevent the rats from being injury. During the test, the time from the rats beginning to grab wire to fall down is recorded. The average forelimb hanging score was recorded according to the hanging time of rats on the wire. In general, the scoring criteria were as follows: 0-5 s: 0 point, 6-10 s: 1 point, 11-15 s: 2 points, 16-20 s: 3 points, 21-25 s: 4 points, 26-30 s: 5 point, and 6 points for more than 30-60 s. Three trials were performed for each rat to obtain the average forelimb hanging score, and the rest time for the rat was about 2 min after a trial. Total three training in 3 independent days were performed for each mouse before the test.

ELISA

After treatment, cell culture mediums were collected and centrifuged at 1000× g for 20 min, and the supernatant were obtained for the measurement of the activity of MAO and M-AChR using the ELISA kit according to the manufacturer's instructions. The colorimetric reading of the solution was determined at OD 450 nm by spectrophotometer (BioTek, VT Lab USA). After calculating the standard curve, the concentrations of MAO and M-AChR were then calculated by the extrapolation from the standard curve.

Statistics

The statistics of data were performed by using GraphPad Prism 8.0 Software (San Diego, CA, USA), and One-way analysis of variance (ANOVA) analysis followed by followed by Tukey's multiple-comparison post hoc test was used for the statistical analysis to compare all the groups. The data were presented as means ± SD, and $P \leq 0.05$ was considered to have statistical significance between the groups.

References

1. Teng JF, *et al.* Polyphyllin VI Induces Caspase-1-Mediated Pyroptosis via the Induction of ROS/NF-kappaB/NLRP3/GSDMD Signal Axis in Non-Small Cell Lung Cancer. *Cancers (Basel)* **12**, (2020).
2. Wu AG, *et al.* Novel steroidal saponin isolated from Trillium tschonoskii maxim. exhibits anti-oxidative effect via autophagy induction in cellular and Caenorhabditis elegans models. *Phytomedicine* **65**, 153088 (2019).
3. Dagda RK, Zhu J, Kulich SM, Chu CT. Mitochondrially localized ERK2 regulates mitophagy and autophagic cell stress: implications for Parkinson's disease. *Autophagy* **4**, 770-782 (2008).
4. Kaizuka T, *et al.* An Autophagic Flux Probe that Releases an Internal Control. *Mol Cell* **64**, 835-849 (2016).
5. An HK, *et al.* CASP9 (caspase 9) is essential for autophagosome maturation through regulation of mitochondrial homeostasis. *Autophagy* **16**, 1598-1617 (2020).
6. Li Q, *et al.* Ochratoxin A causes mitochondrial dysfunction, apoptotic and autophagic cell death and also induces mitochondrial biogenesis in human gastric epithelium cells. *Arch Toxicol* **93**, 1141-1155 (2019).
7. Williams JA, Zhao K, Jin S, Ding WX. New methods for monitoring mitochondrial biogenesis and mitophagy in vitro and in vivo. *Exp Biol Med (Maywood)* **242**, 781-787 (2017).
8. Teng JF, *et al.* Polyphyllin VI, a saponin from Trillium tschonoskii Maxim. induces apoptotic and autophagic cell death via the ROS triggered mTOR signaling pathway in non-small cell lung cancer. *Pharmacol Res* **147**, 104396 (2019).
9. Valero MS, Fagundes DS, Grasa L, Arruebo MP, Plaza MA, Murillo MD. Contractile effect of tachykinins on rabbit small intestine. *Acta Pharmacol Sin* **32**, 487-494 (2011).
10. Palmisano NJ, Melendez A. Detection of Autophagy in Caenorhabditis elegans Using GFP::LGG-1 as an Autophagy Marker. *Cold Spring Harb Protoc* **2016**, pdb prot086496 (2016).
11. Fu RH, *et al.* n-butylidenephthalide protects against dopaminergic neuron degeneration and alpha-synuclein accumulation in Caenorhabditis elegans models of Parkinson's disease. *PLoS One* **9**, e85305 (2014).
12. Fu RH, *et al.* Acetylcorynoline attenuates dopaminergic neuron degeneration and alpha-synuclein aggregation in animal models of Parkinson's disease. *Neuropharmacology* **82**, 108-120 (2014).
13. Fu RH, *et al.* n-Butylidenephthalide Protects against Dopaminergic Neuron Degeneration and α-Synuclein Accumulation in Caenorhabditis elegans Models of Parkinson's Disease. *Plos One* **9**, e85305- (2014).
14. Bodhicharla R, *et al.* Effects of alpha-synuclein overexpression in transgenic Caenorhabditis elegans strains. *CNS Neurol Disord Drug Targets* **11**, 965-975 (2012).
15. Borlongan CV, Randall TS, Cahill DW, Sanberg PR. Asymmetrical motor behavior in rats with unilateral striatal excitotoxic lesions as revealed by the elevated body swing test. *Brain Res* **676**, 231-234 (1995).
16. Haobam R, Sindhu KM, Chandra G, Mohanakumar KP. Swim-test as a function of motor impairment in MPTP model of Parkinson's disease: a comparative study in two mouse strains. *Behav Brain Res* **163**, 159-167 (2005).
17. Wu AG, *et al.* Hederagenin and alpha-hederin promote degradation of proteins in neurodegenerative diseases and improve motor deficits in MPTP-mice. *Pharmacol Res* **115**, 25-44 (2017).

18. Zhang L, Haraguchi S, Koda T, Hashimoto K, Nakagawara A. Muscle atrophy and motor neuron degeneration in human NEDL1 transgenic mice. *J Biomed Biotechnol* **2011**, 831092 (2011).

Supplemental Figures

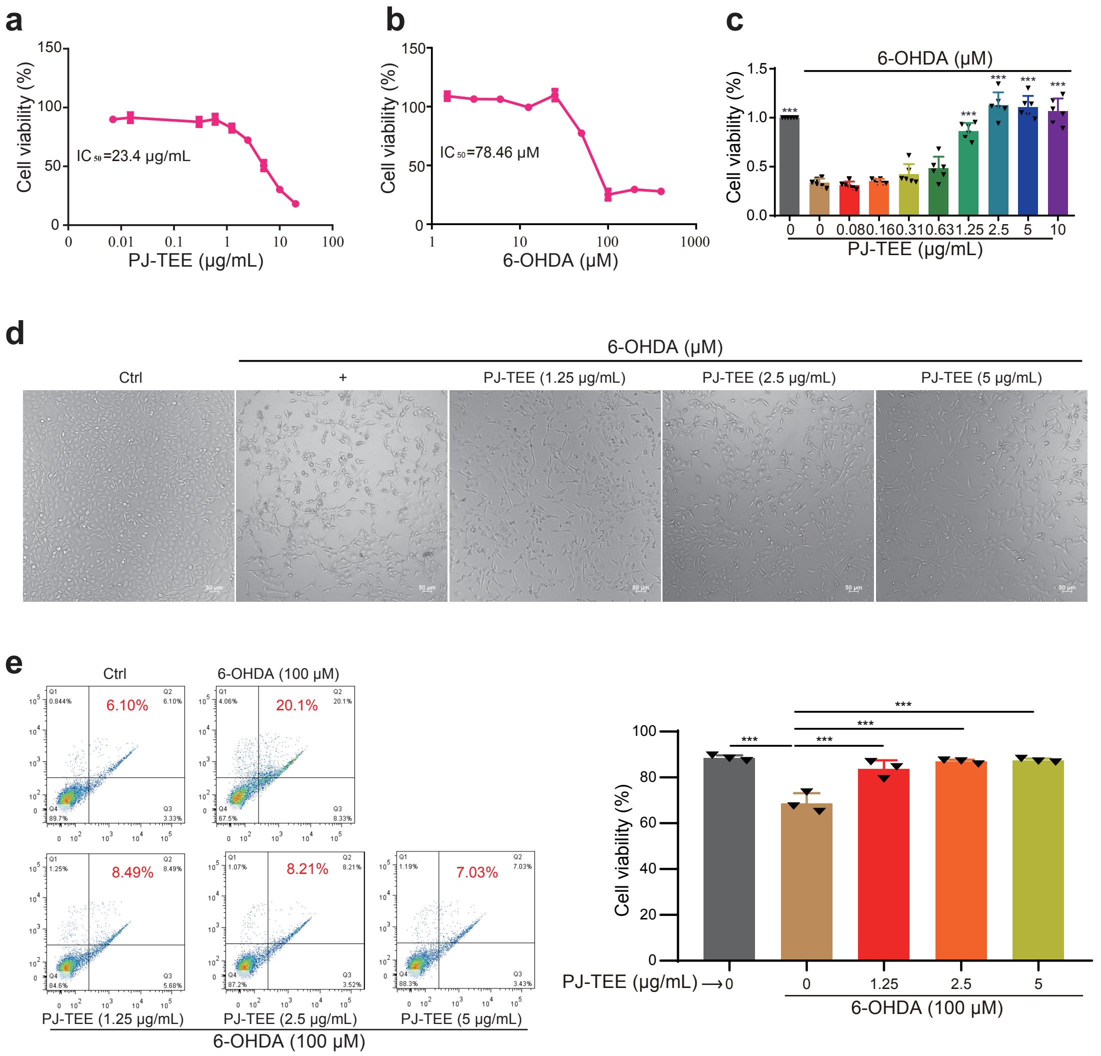

Supplementary Fig. 1 PJ-TEE protects against 6-OHDA-induced damage in PC-12 cells. a Cytotoxicity of PJ-TEE in PC-12 cells after 48 h of treatment was determined by MTT assay. The IC_{50} value of PJ-TEE were calculated by GraphPad Prism 8.0 Software (San Diego, CA, USA). **b** Cytotoxicity of 6-OHDA in PC-12 cells after 48 h of treatment was determined by MTT assay. The IC_{50} value of 6-OHDA was calculated by using GraphPad Prism 8.0 Software. **c** 6-OHDA-induced PC-12 cells were treated with PJ at the indicated concentrations for 24 h. After treatment, the cell viability of PC-12 cells was determined by MTT assay. Bar chart indicated the relative percentage of cell viability of PC-12 cells. Bars, S.D., *** $p \leq 0.001$. **d** 6-OHDA-induced PC-12 cells were treated with PJ at the indicated concentrations for 24 h. After treatment, the representative images of PC-12 cells were captured by microscope (magnification, ×10,

scale bar: 200 μM). **e** 6-OHDA-induced PC-12 cells were treated with PJ at the indicated concentrations for 24 h. After treatment, the cell apoptosis of PC-12 cells was analyzed by flow cytometry. Bar chart indicated the viability of PC-12 cells. Bars, S.D., ** $p \leq 0.01$. Data were obtained from 3 independent experiments.

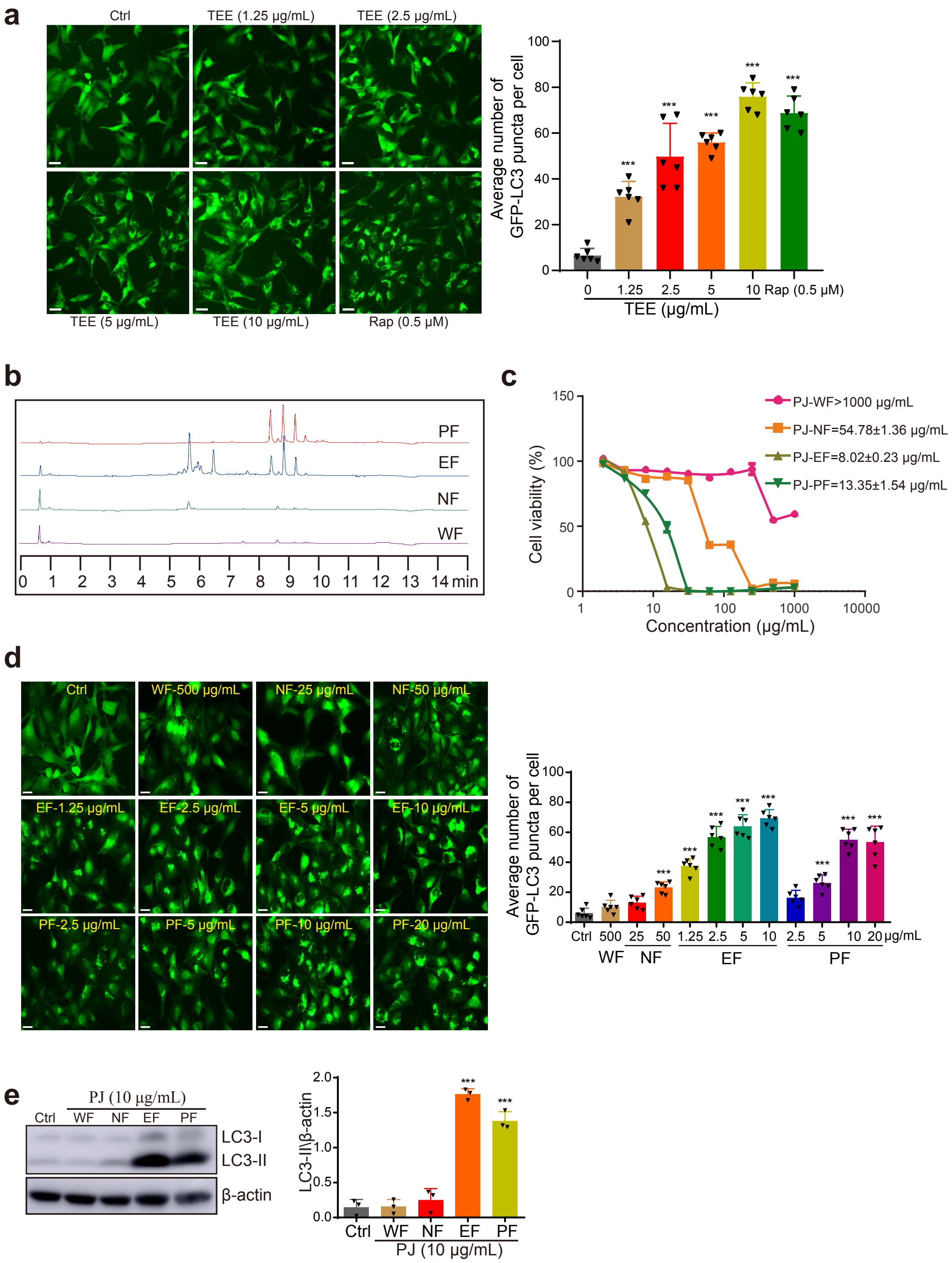

Supplementary Fig. 2 EF isolated from PJ-TEE induces the highest autophagic activity. a Stable RFP-GFP-LC3 U87 cells were treated with PJ-TEE at the indicated concentrations for 24 h. After treatment, cells were fixed by 4% PFA and the representative images were captured by fluorescence microscope (magnification, ×40,

scale bar: 50 μm). Bar chart indicated the percentage of cells with GFP-LC3 puncta formation. Bars, S.D., *** $p \leq 0.001$. **b** The total ion chromatogram (TIC) of WF, NF, EF, and PF. All samples were separated on an Agilent Zorbax Eclipse Plus C18 (100 mm × 2.1 mm) column and analyzed by UHPLC-DAD-TOF/MS in positive mode. **c** Cytotoxicity of WF, NF, EF, and PF in stable RPF-GFP-LC3 U87 cells after 48 h of treatment was determined by MTT assay. The IC_{50} values were calculated by GraphPad Prism 8.0 Software. **d** Stable RPF-GFP-LC3 U87 cells were treated with WF, NF, EF or PF at the indicated concentrations for 24 h. After treatment, cells were fixed by 4% PFA and the representative images were captured by a fluorescence microscope (magnification, ×40, scale bar: 50 μm). Bar chart indicated the percentage of cells with GFP-LC3 puncta formation. Bars, S.D., *** $p \leq 0.001$. **e** PC-12 cells were treated with 10 μg/mL of WF, NF, EF or PF for 24 h. After treatment, cells lysates were harvested for the analysis of LC3 and β-actin using Western blot. Bar chart indicated the relative protein expression of LC3-II to β-actin. Bars, S.D., *** $p \leq 0.001$. The full-length western blotting images were showed in **Supplementary Fig. 26**.

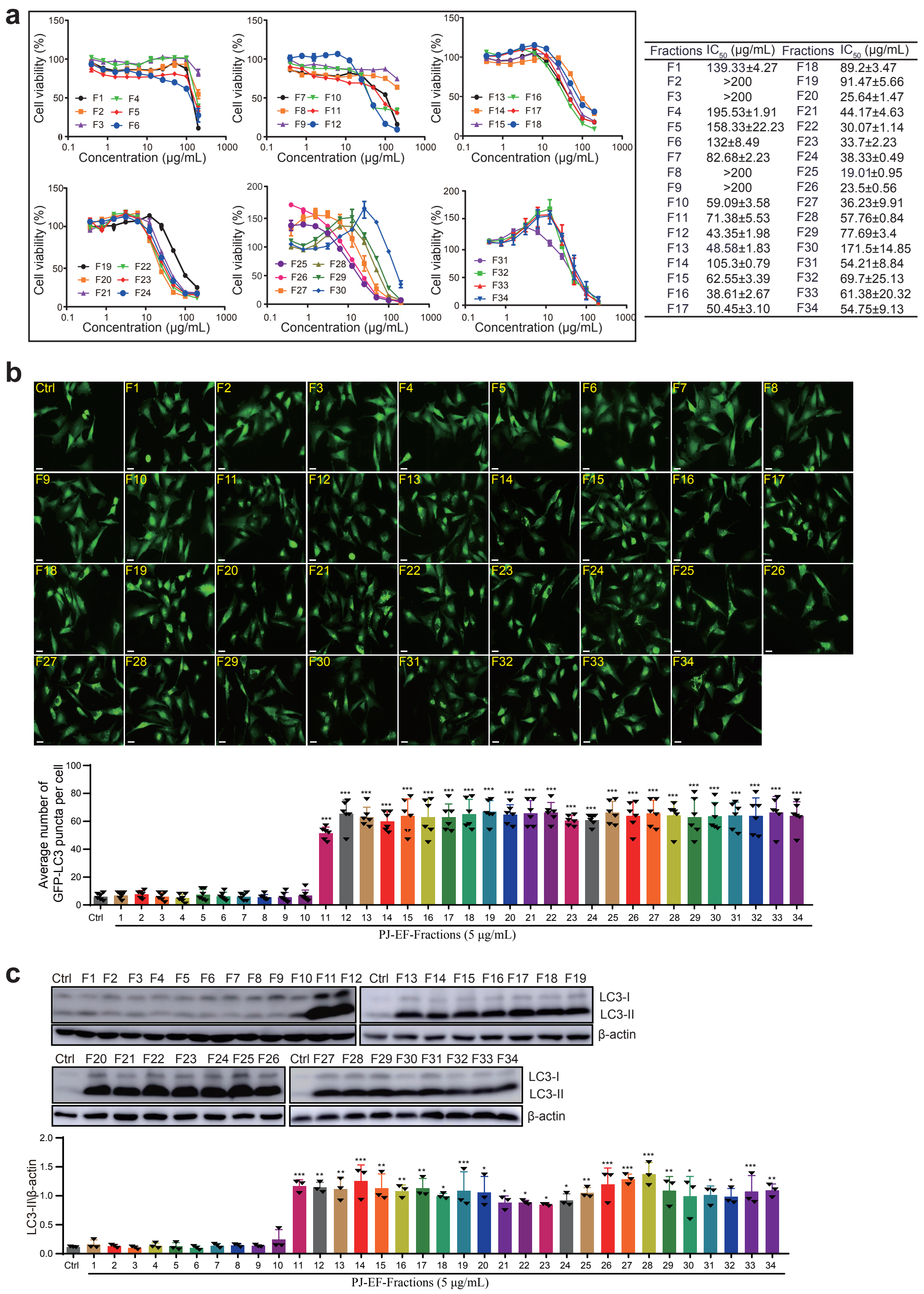

Fractions	IC_{50} (μg/mL)	Fractions	IC_{50} (μg/mL)
F1	139.33±4.27	F18	89.2±3.47
F2	>200	F19	91.47±5.66
F3	>200	F20	25.64±1.47
F4	195.53±1.91	F21	44.17±4.63
F5	158.33±22.23	F22	30.07±1.14
F6	132±8.49	F23	33.7±2.23
F7	82.68±2.23	F24	38.33±0.49
F8	>200	F25	19.01±0.95
F9	>200	F26	23.5±0.56
F10	59.09±3.58	F27	36.23±9.91
F11	71.38±5.53	F28	57.76±0.84
F12	43.35±1.98	F29	77.69±3.4
F13	48.58±1.83	F30	171.5±14.85
F14	105.3±0.79	F31	54.21±8.84
F15	62.55±3.39	F32	69.7±25.13
F16	38.61±2.67	F33	61.38±20.32
F17	50.45±3.10	F34	54.75±9.13

Supplementary Fig. 3 F11-F34 isolated from EF of PJ-TEE induce autophagy. a Cytotoxicity of F1-F34 in stable RPF-GFP-LC3 U87 cells after 48 h of treatment was determined by MTT assay. The IC_{50} values of F1-F34 showed in the table were calculated by GraphPad Prism 8.0 Software. **b** Stable RPF-GFP-LC3 U87 cells were treated with F1-F34 at the indicated concentrations for 24 h. After treatment, cells were fixed by 4% PFA and the representative images were captured by a fluorescence

microscope (magnification, ×40, scale bar: 50 μm). Bar chart indicated the percentage of cells with GFP-LC3 puncta formation. Bars, S.D., *** $p \leq 0.001$. **c** PC-12 cells were treated with 5 μg/mL of F1-F34 for 24 h. After treatment, cells lysates were harvested for the analysis of LC3 and β-actin using Western blot. Bar chart indicated the relative protein expression of LC3-II to β-actin. Bars, S.D., * $p \leq 0.05$, ** $p \leq 0.01$, *** $p \leq 0.001$. The full-length western blotting images were showed in **Supplementary Fig. 26**.

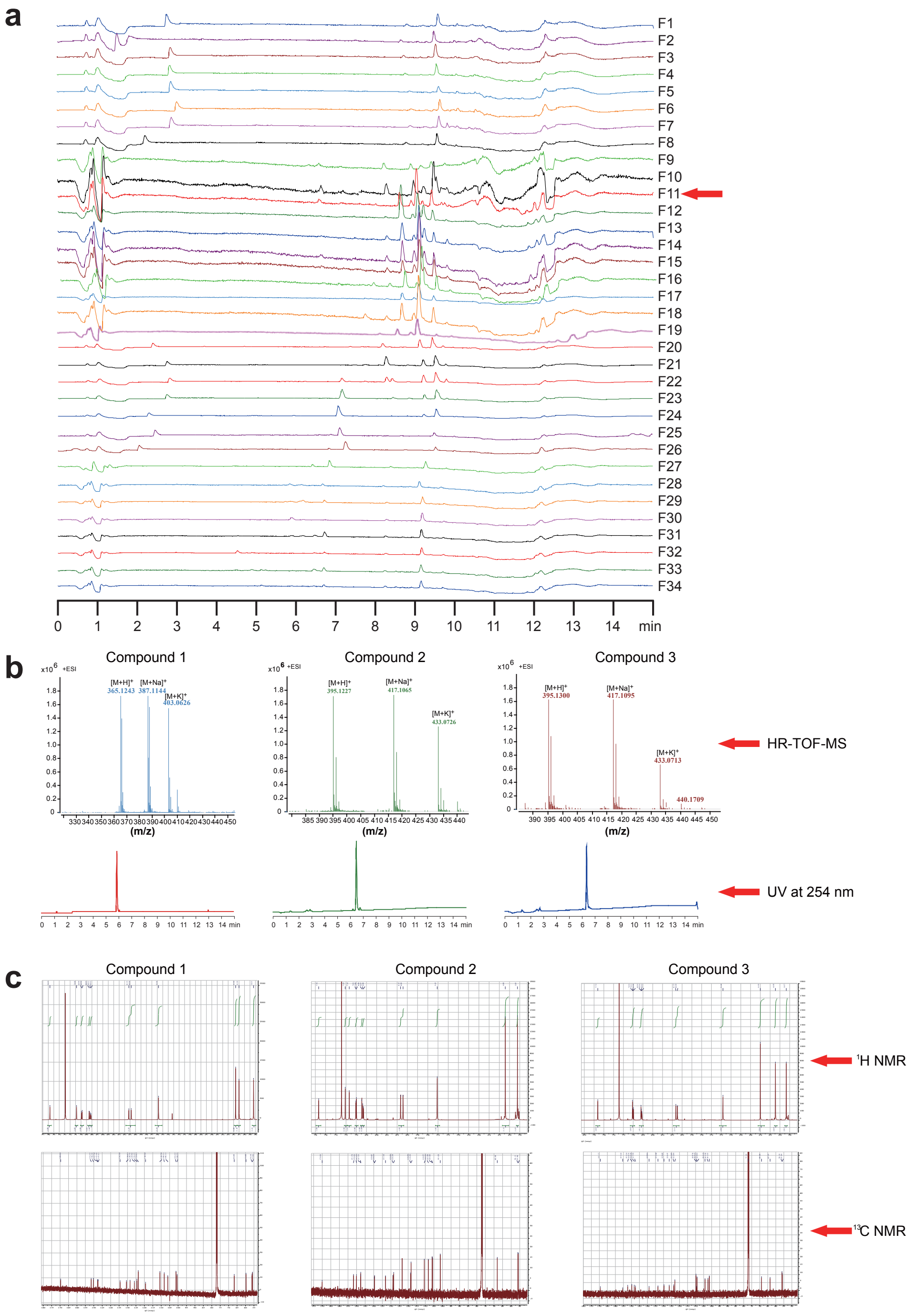

Supplementary Fig. 4 Isolation and elucidation of JA, JB and JC. a TIC of F1-F34 isolated from EF of PJ-TEE. **b** The mass spectrums and UHPLC-UV chromatograms

at 254 nm of compound 1, compound 2 and compound 3. All samples were separated on the Agilent Zorbax Eclipse Plus C18 (100 mm × 2.1 mm) column and analyzed by UHPLC-DAD-TOF/MS in positive mode. **c** The ^{1}H-NMR spectrum and ^{13}C-NMR spectrum of JA, JB and JC.

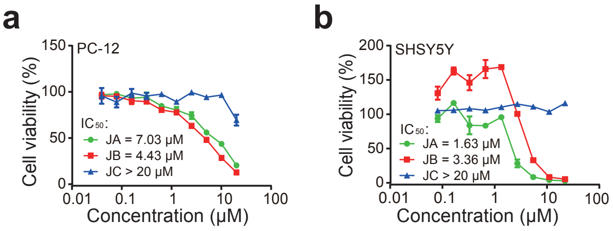

Supplementary Fig. 5 Cytotoxicity of JA, JB and JC in PC-12 cells (a) or SHSY5Y cells (b) after 48 h of treatment were determined by MTT assay. The IC_{50} values of JA, JB and JC were calculated by GraphPad Prism 8.0 Software.

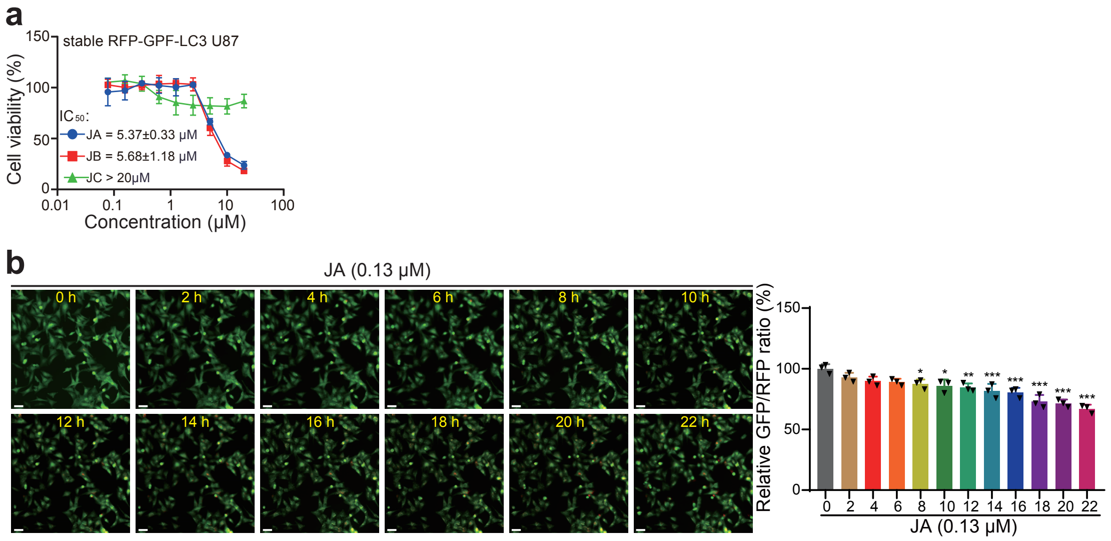

Supplementary Fig. 6 JA increases autophagic flux in stable RFP-GFP-LC3 U87 cells. **a** Cytotoxicity of JA, JB and JC in stable RFP-GFP-LC3 U87 cells after 48 h of treatment was determined by MTT assay. The IC_{50} value of JA, JB and JC were calculated by GraphPad Prism 8.0 Software. **b** The time-lapse phase-contrast and fluorescent images of stable RFP-GFP-LC3 U87 cells were captured at the indicated timepoints after the stimulated by 0.13 μM of JA. The images captured under the red or green channels were merged at the same field. The real-time video was included in **Supplementary Video 1**. Bar chart indicated the relative ratio of GFP/RFP; bars, S.D. * $p \leq 0.05$, ** $p \leq 0.01$, *** $p \leq 0.001$. Data shown were representatives of at least 3 independent experiments. Magnification: 10×, Scale bar: 200 μm.

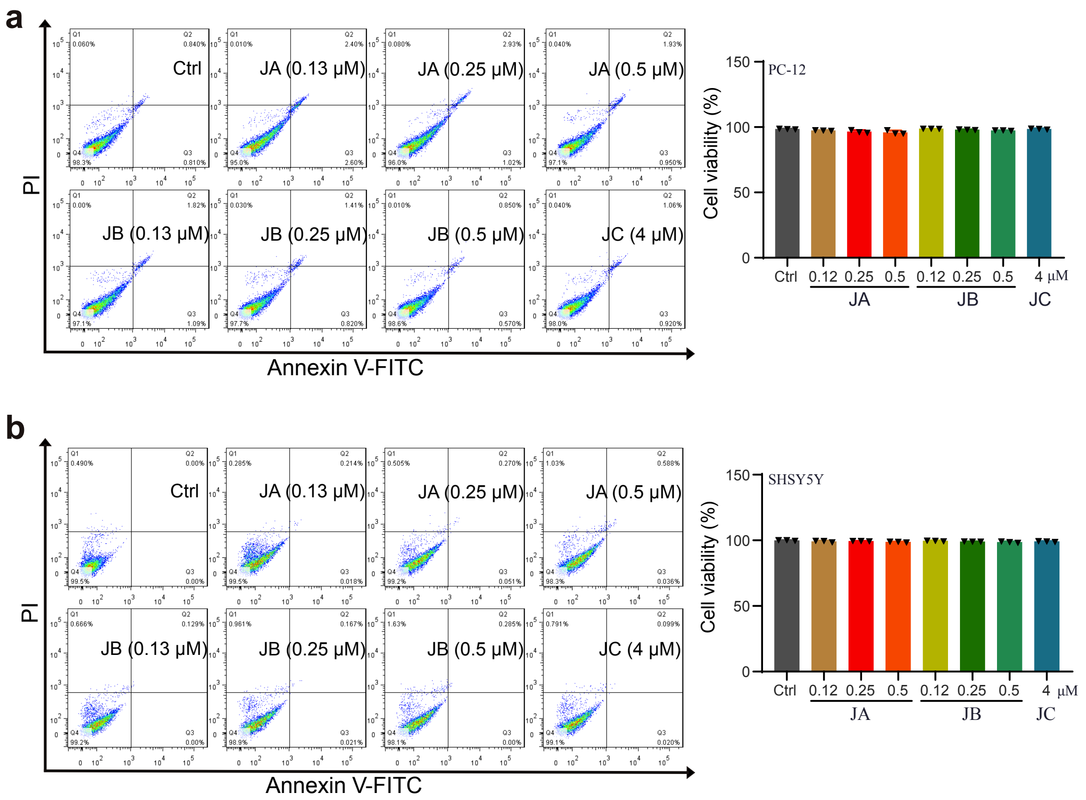

Supplementary Fig. 7 Cell viability of JA, JB and JC in PC-12 cells (a) or SHSY5Y cells (b) after 24 h of treatment were determined by a flow cytometer using the Annexin V-FITC/PI Apoptosis detection Kit. Bar chart indicated the cell viability of PC-12 cells and SHSY5Y cells.

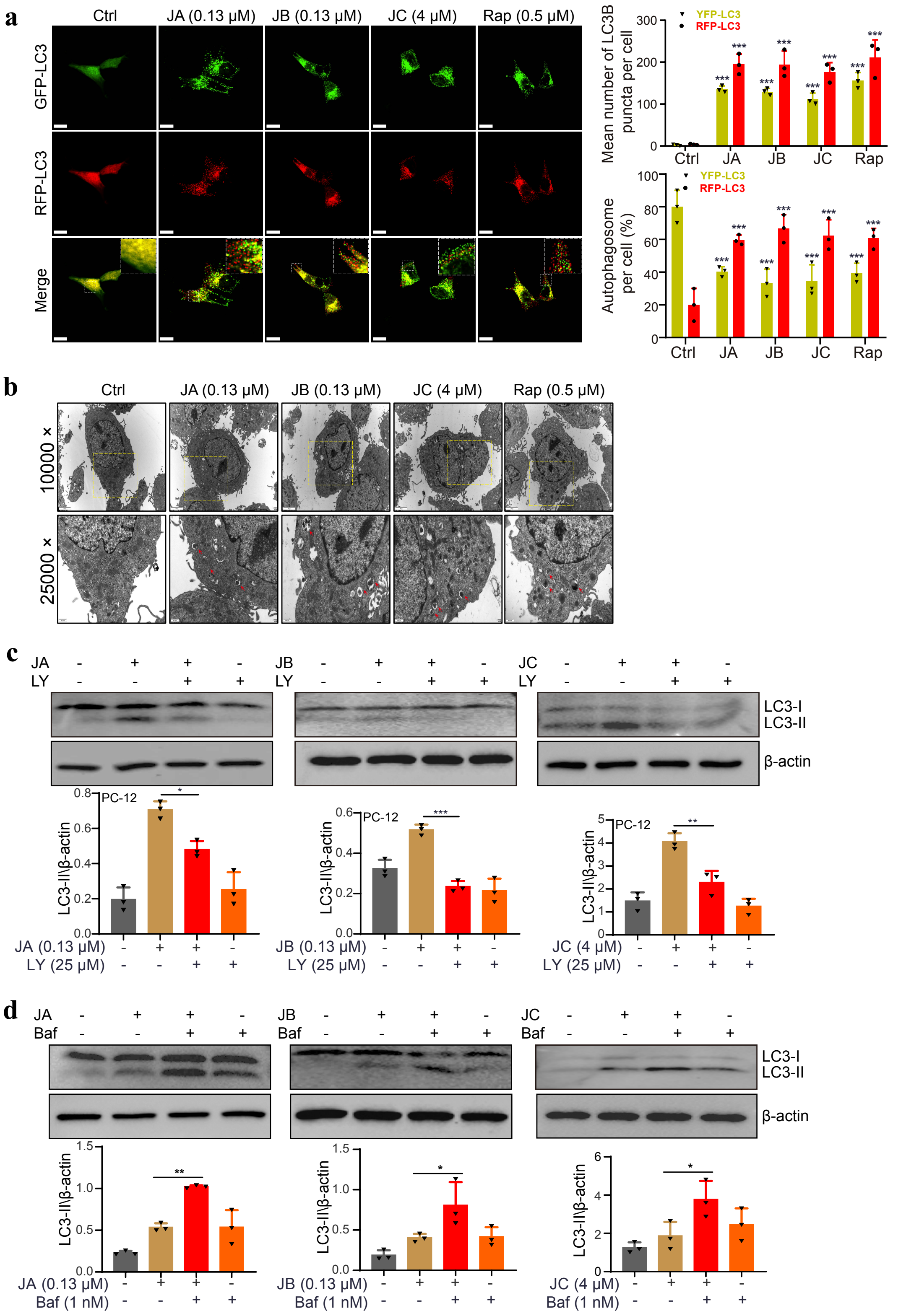

Supplementary Fig. 8 JA, JB and JC induce autophagy flux in PC-12 cells. a PC-12 cells were transiently transfected with tfLC3 plasmid and then incubated with JA, JB, JC, or Rap under the indicated concentrations for 24 h. Representative cell images with GFP-LC3 and RFP-LC3 were captured by a Leica SP8 confocal microscope (magnification, 63 ×). Scale bar: 5 μm. Bar chart indicated the mean number of LC3B

puncta per cell and the percentage of autophagosome per cell. Bars, S.D., *** $p \leq 0.001$. **b** Representative electron micrographs showing the ultra-structures of PC-12 cells were treated with JA (0.13 μM), JB (0.13 μM), JC (4 μM) or Rap (0.5 μM) for 24 h. Upper: Magnification: ×10000, scale bar: 2 μm (**upper**); ×250000, scale bar: 500 nm (**bottom**). Red arrows, double-membraned autophagosomes. **c** PC-12 cells or (**d**) SHSY5Y cells were treated with JA, JB or JC in the presence or absence of LY or Baf at the indicated concentrations for 24 h. Cells were then harvested and the proteins were analyzed for the expression of LC3 and β-actin using Western blotting. Bar chart indicated the expression of LC3-II/β-actin in PC-12 cells under the indicated treatments. Bars, S.D., * $p \leq 0.05$, ** $p \leq 0.01$, *** $p \leq 0.001$. The full length of western blotting images was showed in the **Supplementary Fig. 28**.

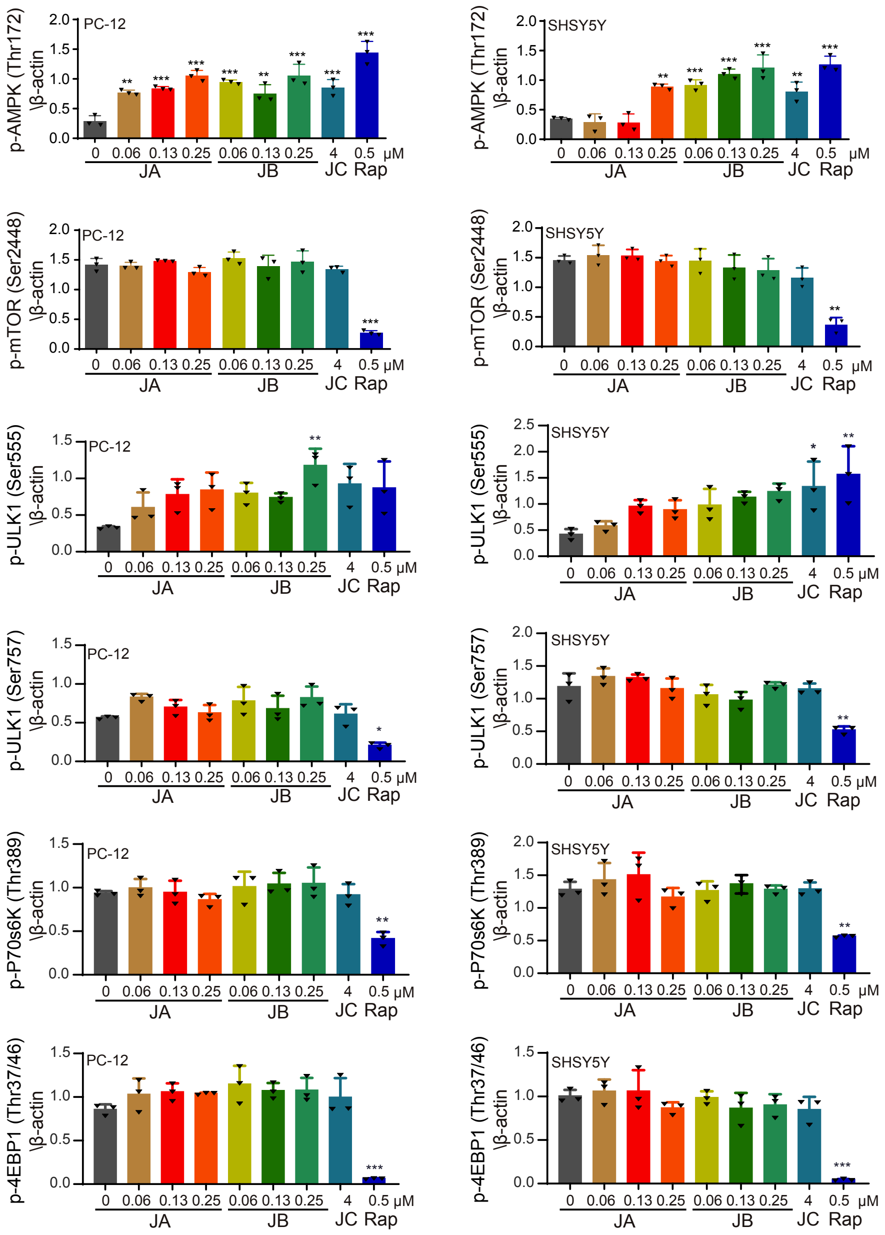

Supplementary Fig. 9 The relative protein expression of p-AMPK (Thr172), p-mTOR (Ser2448), p-ULK1 (Ser555), p-ULK1 (Ser757), p-P70s6K (Thr389) or p-4EBP1 (Thr37/46) to β-actin in PC-12 cells and SHSY5Y cells. Bars, S.D., * $p \leq 0.05$, ** $p \leq 0.01$, *** $p \leq 0.001$

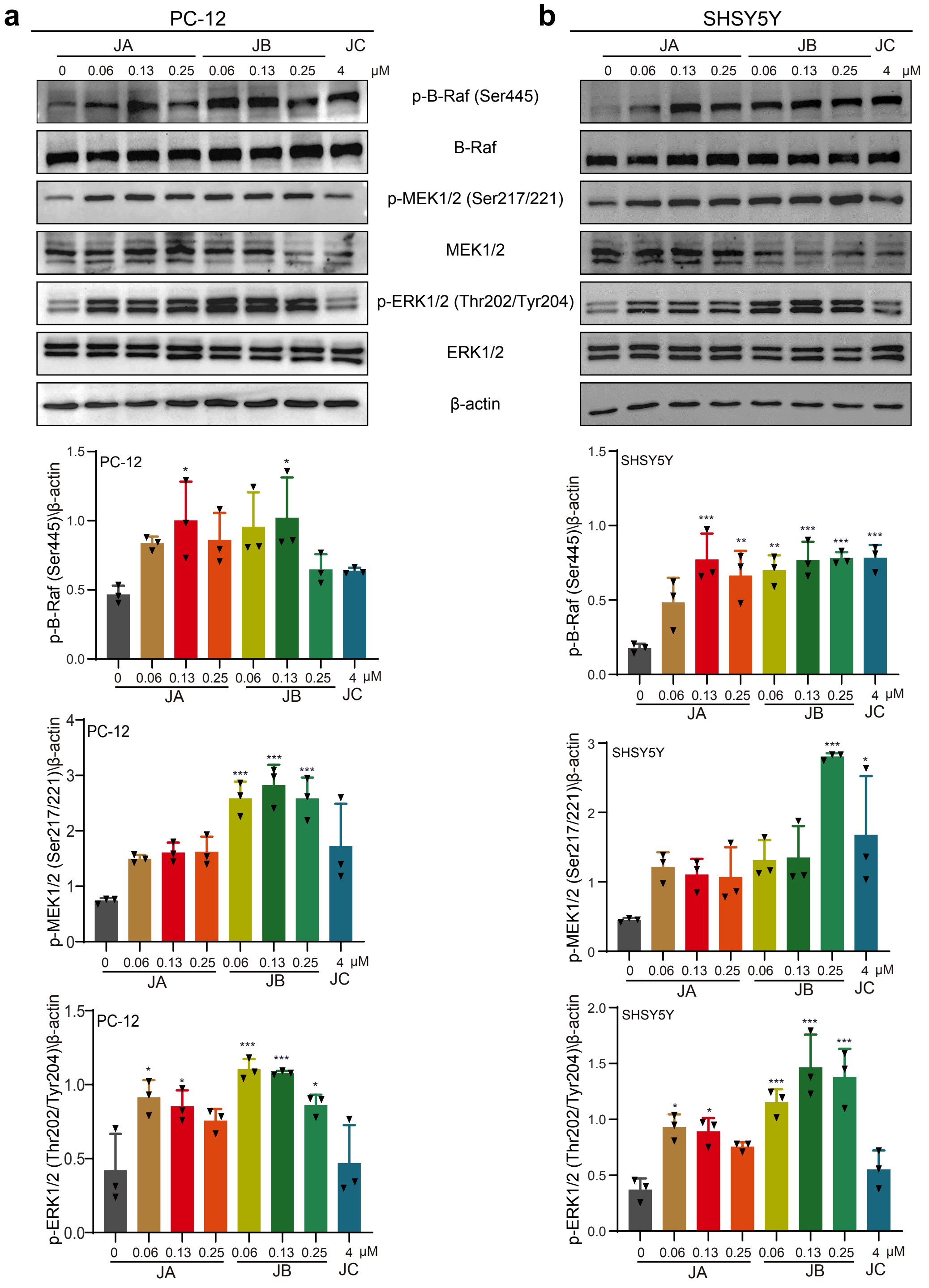

Supplementary Fig. 10 JA, JB and JC activate the Raf/MEK/ERK signaling pathway. (a) PC-12 cells or **(b)** SHSY5Y cells seeded on the 6-well plates were treated with JA, JB and JC at the indicated concentrations for 24 h. After treatment, cell proteins were collected for the Western blotting analysis of p-B-Raf (Ser445), B-Raf,

p-MEK1/2 (Ser217/221), MEK1/2, p-ERK1/2 (Thr1/2), ERK1/2 and β-actin. Bar chart indicated the relative expression of the interest protein to β-actin. Bars, S.D., * $p \leq 0.05$, ** $p \leq 0.01$, *** $p \leq 0.001$. The full length of western blotting images was showed in the **Supplementary Fig. 30**.

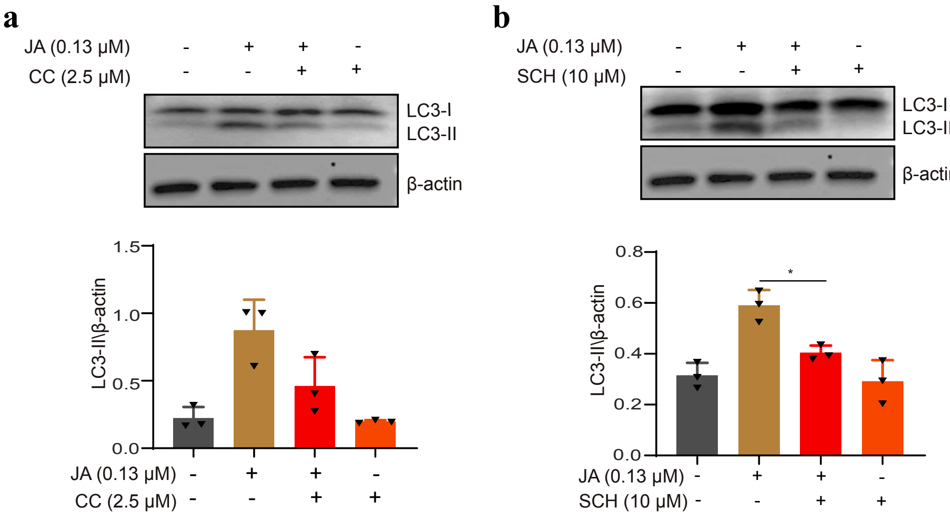

Supplementary Fig. 11 Compound C (CC) and SCH772984 (SCH) inhibit JA-induced LC3-II expression in PC-12 cells. PC-12 cells seeded on the 6-well plates were treated with JA, in the presence or absence of CC (**a**) or SCH (**b**) at the indicated concentrations for 24 h. After treatment, cell proteins were collected for the Western blotting analysis of LC3 and β-actin. Bar chart indicated the relative expression of LC3-II to β-actin. Bars, S.D., * $p \leq 0.05$. The full length of western blotting images was showed in the **Supplementary Fig. 31**.

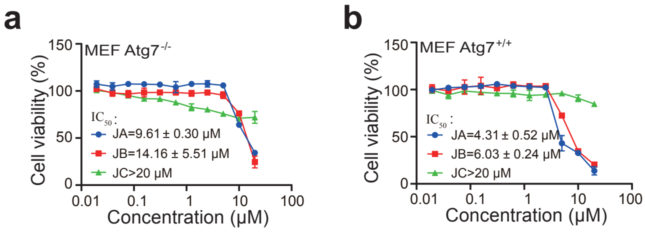

Supplementary Fig. 12 Cytotoxicity of JA, JB and JC in Atg7-knockout MEF cells (a) and wild type MEF cells (b) after 48 h of treatment were determined by MTT assay. The IC_{50} values of JA, JB and JC were calculated by GraphPad Prism 8.0 Software.

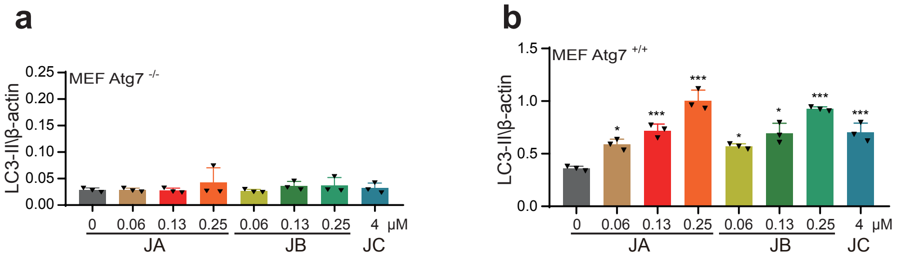

Supplementary Fig. 13 The relative protein expression of LC3-II to β-actin in Atg7-knockout MEF cells (**a**) and wild type MEF cells (**b**). Bars, S.D., * $p \leq 0.05$, ** $p \leq 0.01$, *** $p \leq 0.001$.

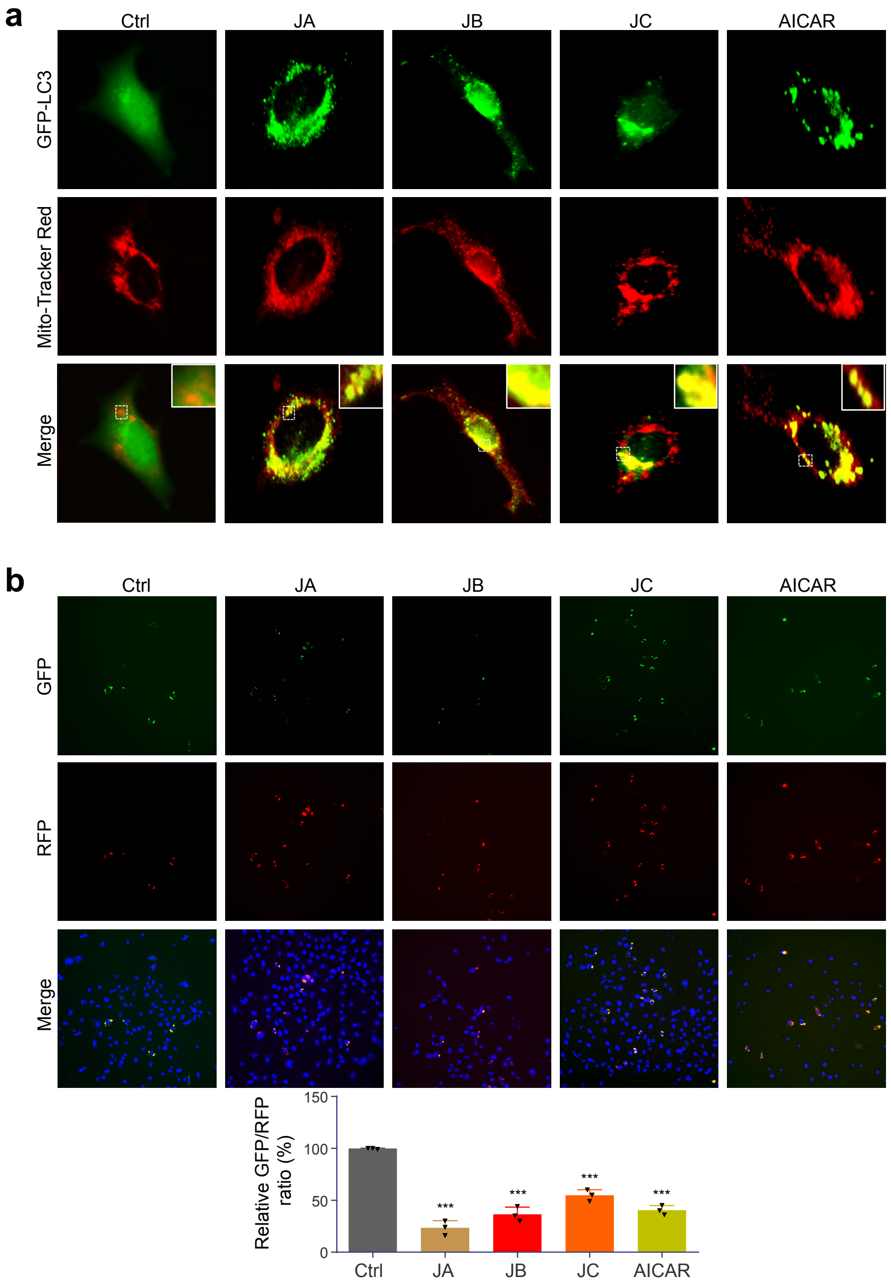

Supplementary Fig. 14 JA, JB and JC activate mitophagy in PC-12 cells. a PC-12

cells seeded on 6-well plates were transiently transfected with GFP-LC3 plasmid for 24 h, and followed by the treatment of JA (0.13 μM), JB (0.13 μM), JC (4 μM) or AICAR (250 μM) for 24 h. Then, cells were incubated with Mito-Tracker Red CMXRos solution for 30 min at 37 °C. After incubation, the Mito-Tracker Red CMXRos solution was exchanged by the fresh cell culture medium, and colocalization of GFP-LC3 and Mito-Tracker Red was visualized under the fluorescence microscope (100× magnification. Scale bar: 10 μm). **b** PC-12 cells seeded on 6-well plates were transiently transfected with mito-QC plasmid for 24 h, and followed by the treatment of JA (0.13 μM), JB (0.13 μM), JC (4 μM) or AICAR (250 μM) for 24 h. After treatment, cells were examined and the representative images were captured by a fluorescence microscope (10× magnification, Scale bar: 50 μm). Bar chart indicated the relative GFP/RFP ratio. Bars, S.D., *** $p \leq 0.001$.

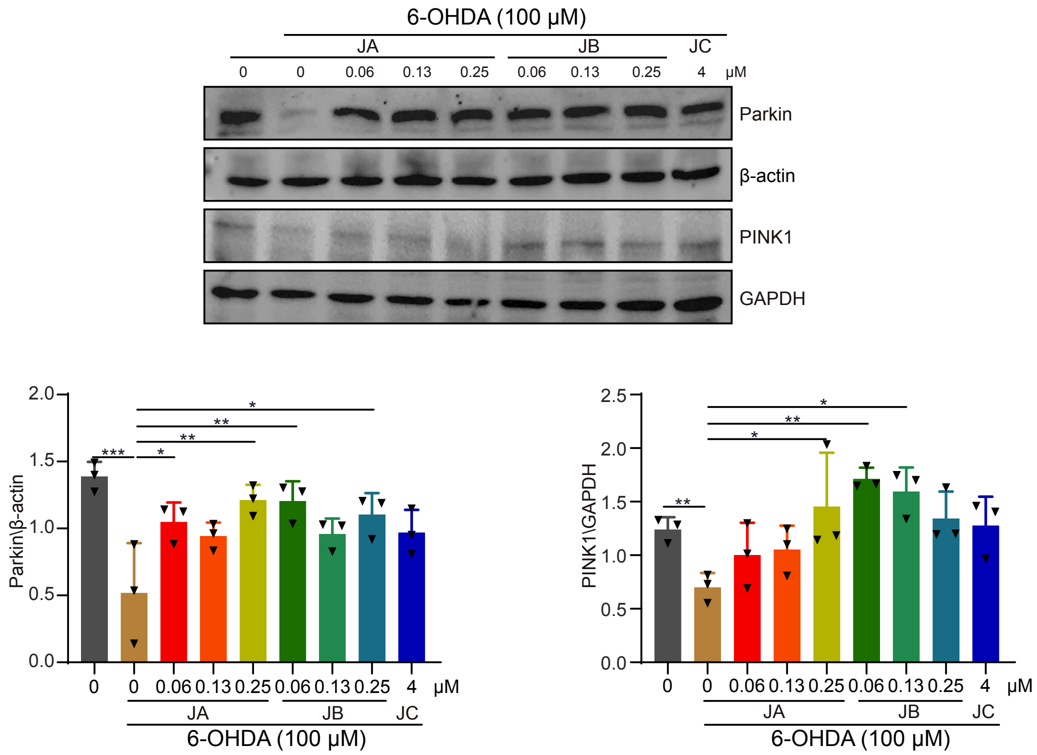

Supplementary Fig. 15 JA, JB and JC activate the Parkin/PINK1 signaling pathway. PC-12 cells seeded on the 6-well plates were treated with JA, JB and JC at the indicated concentrations for 24 h. After treatment, cell proteins were collected for the Western blotting analysis of Parkin, PINK, β-actin and GAPDH. Bar chart indicated the relative expression of the Parkin to β-actin or PINK1/GAPDH. Bars, S.D., * $p \leq 0.05$, ** $p \leq 0.01$, *** $p \leq 0.001$. The full length of western blotting images was showed in the **Supplementary Fig. 33**.

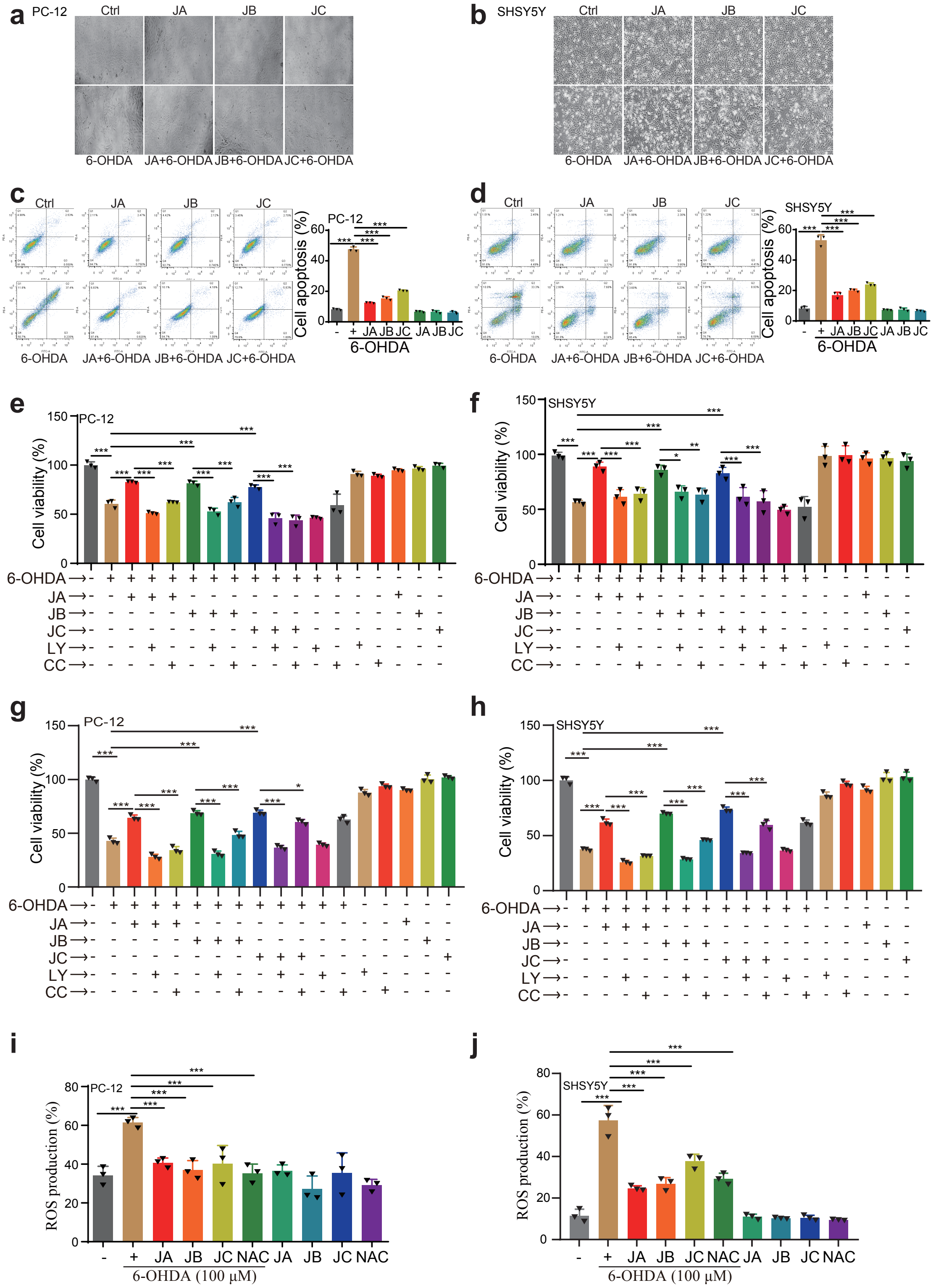

Supplementary Fig. 16 JA, JB and JC increase the viability of 6-OHDA-induced PC-12 cells and SHSY5Y cells via autophagy induction. PC-12 cells (**a**) or SHSY5Y cells (**b**) were induced by 6-OHDA (100 μM) and treated with JA (0.13 μM), JB (0.13 μM) or JC (4 μM) for 24 h. After treatment, the representative images of cell morphology were captured by a microscope with 10× magnification. Scale bar: 50 μm.

PC-12 cells (**c**) or SHSY5Y cells (**d**) were induced by 6-OHDA (100 μM) and treated with JA (0.13 μM), JB (0.13 μM) or JC (4 μM) for 24 h. After treatment, cells were analyzed by a flow cytometer using the FITC/PI apoptotic detection kit. Bar chart indicated the percentage of apoptotic cells. Bars, S.D., *** $p \leq 0.001$. PC-12 cells (**e**) or SHSY5Y cells (**f**) were induced by 6-OHDA (100 μM) and treated with JA (0.13 μM), JB (0.13 μM) or JC (4 μM) in the absence or presence of LY (25 μM) or CC (2.5 μM) for 24 h. After treatments, the viability of PC-12 cells or SHSY5Y cells was measured by MTT assay. Bar chart indicated the relative percentage of cell viability. Bars, S.D., * $p \leq 0.05$, ** $p \leq 0.01$, *** $p \leq 0.001$. PC-12 cells (**g**) or SHSY5Y cells (**h**) were induced by 6-OHDA (100 μM) and treated with JA (0.13 μM), JB (0.13 μM) or JC (4 μM) in the absence or presence of LY (25 μM) or CC (2.5 μM) for 24 h. After treatments, the viability of PC-12 cells or SHSY5Y cells was measured by CCK8 assay. Bar chart indicated the relative percentage of cell viability. Bars, S.D., * $p \leq 0.05$, *** $p \leq 0.001$. Statistics analysis of ROS production for **Fig. 1h**. Bar chart indicated the percentage of ROS production in PC-12 cells (**i**) or SHSY5Y cells (**j**). Bars, S.D., *** $p \leq 0.001$.

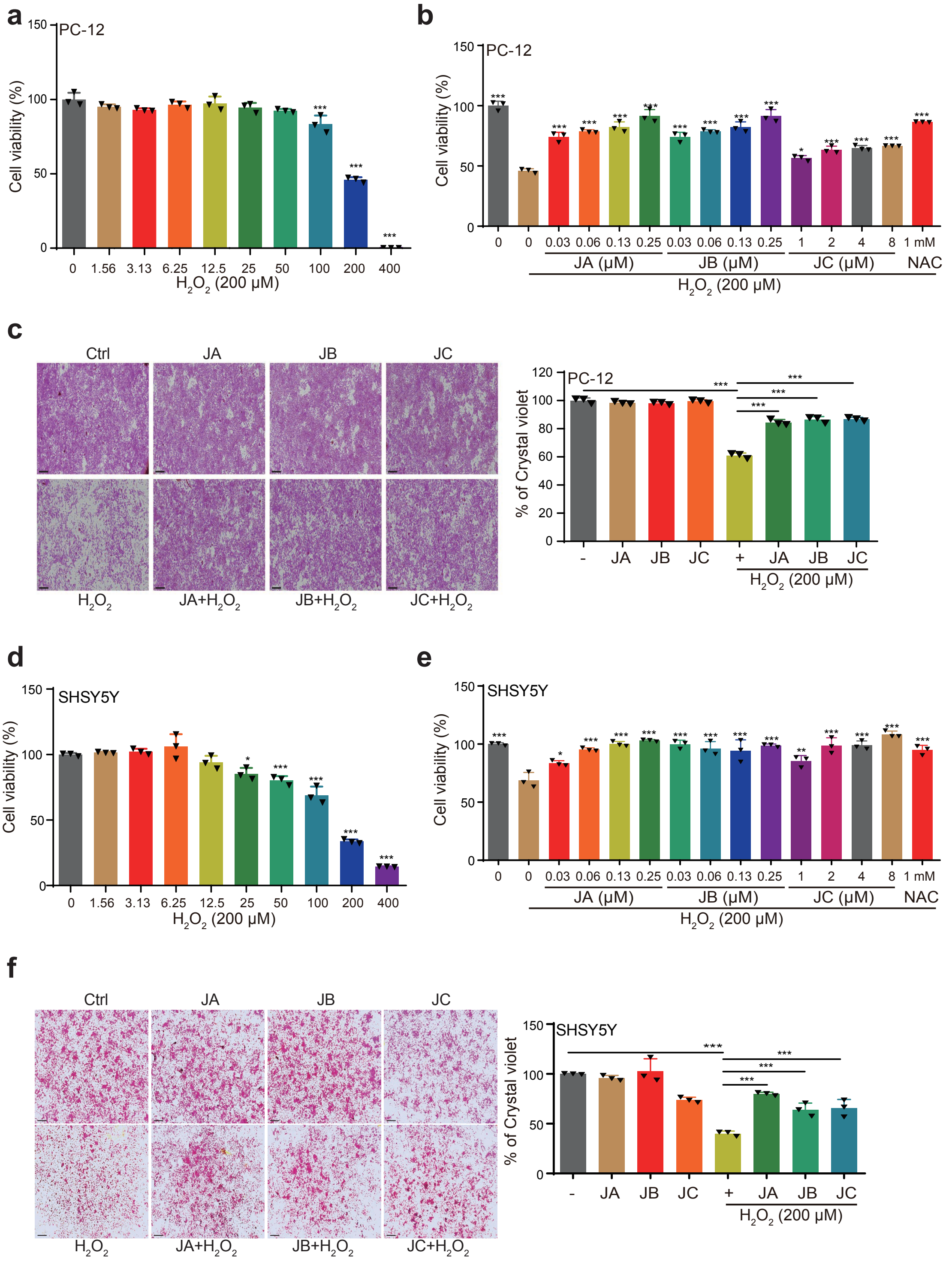

Supplementary Fig. 17 JA, JB and JC rescue PC-12 cells or SHSY5Y cells from cell death induced by H_2O_2. PC-12 cells (**a**) or SHSY5Y cells (**d**) were treated with H_2O_2 at the indicated concentrations for 48 h. After treatments, the cytotoxicity was measured by MTT assay. Bar chart indicated the cell viability of PC-12 or SHSY5Y. Bars, S.D., * $p \leq 0.05$, *** $p \leq 0.001$. H_2O_2-induced PC-12 cells (**b**) or SHSY5Y cells (**e**) were treated with JA, JB and JC at the indicated concentrations for 48 h. After treatment, the cytotoxicity was measured by MTT assay. Bar chart indicated the

viability of PC-12 cells or SHSY5Y cells. Bars, S.D., * $p \leq 0.05$, ** $p \leq 0.01$, *** $p \leq 0.001$. H_2O_2-induced PC-12 cells (**c**) or SHSY5Y cells (**f**) were treated with JA (0.13 μM), JB (0.13 μM) and JC (4 μM) at the indicated concentrations for 24 h. After treatment, the cells were fixed by 4% PFA and stained with crystal violet solution for 30 min. The representative images were captured by a microscope (magnification, ×10, scale bar: 200 μm). Bar chart indicated the relative percentage of crystal violet. Bars, S.D., *** $p \leq 0.001$.

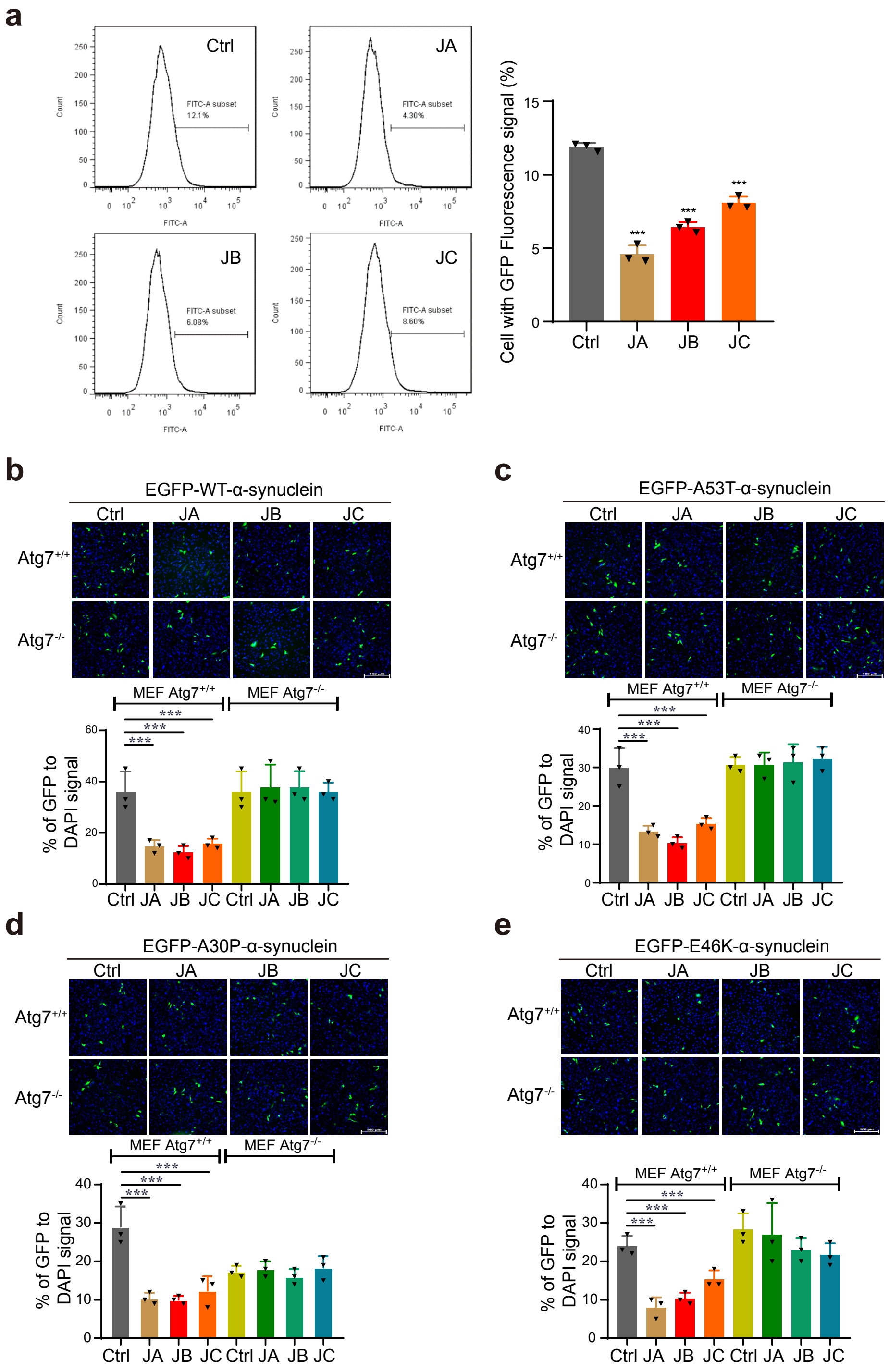

Supplementary Fig. 18 JA, JB and JC inhibit the oligomerization of α-synuclein

in PC-12 cells and decreased the levels of mutantα-synuclein in MEF cells via Atg7. **a** PC-12 cells transfected with both GNS and SGC plasmids were treated with JA (0.13 μM), JB (0.13 μM) or JC (4 μM). After treatment, cells were analyzed by flow cytometer using the FITC channel. Bar chart indicated the percentage of cells with GFP-positive signal. Bars, S.D., *** $p \leq 0.001$. **b**, **c**, **d**, **e** Wild type Atg7 and Atg7-knonckout MEF cells transiently transfected with EGFP-WT-α-synuclein, EGFP-A53T-α-synuclein EGFP-A30P-α-synuclein or EGFP-E46K-α-synuclein were treated with JA (0.13 μM), JB (0.13 μM) or JC (4 μM) for 24 h. After treatment, cells were analyzed and the representative images were captured by the fluorescence microscope (magnification, ×10, scale bar: 100 μm). Bar chart indicated the relative percentage of GFP/DAPI signal. Bars, S.D., *** $p \leq 0.001$.

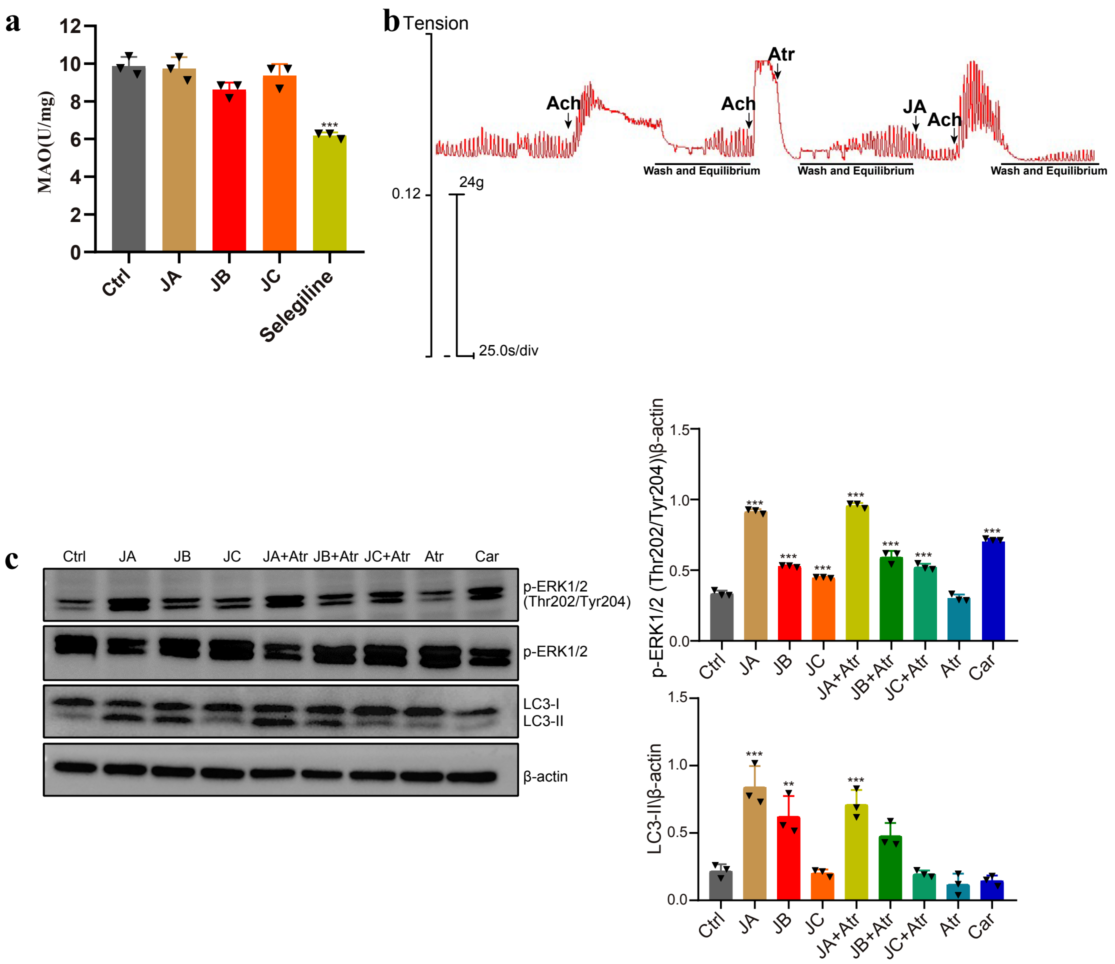

Supplementary Fig. 19 The effect of JA, JB and JC on the activity of MAO and the activation of M receptor. a PC-12 cells were treated with JA (0.13 μM), JB (0.13 μM), JC (4 μM), or selegiline (100 μM) for 24 h. After treatment, cells were collected, homogenized, and centrifugated. Then, the supernatant was collected for the determination of MAO activity by the MAO detection kit according to the

manufacturer's instructions. Bar chart indicated the activity of MAO. Bars, S.D., *** $p \leq 0.001$. **b** Effect of JA on the activation of M receptor. The segment of jejunum was taken from a male New Zealand rabbit and suspended in the direction of longitudinal and circular smooth muscle fibers in a thermostatically controlled (37 °C) organ bath (10 mL capacity) containing Krebs solution. The drugs, including Ach, Atr, and JA, were added into the Krebs solution. The output waveform signals indicated the contraction or relaxation of intestinal muscle. **c** PC-12 cells were treated with carbachol (200 μM), or JA (0.13 μM), JB (0.13 μM), and JC (4 μM) in the presence or absence of Atr (100 μM) for 24h. After treatment, cell lysates were collected for the Western blot analysis of ERK1/2, LC3 and β-actin. Bar chart indicated the relative density of p-ERK1/2 (Thr202/Tyr204) or LC3-II to β-actin. Bars, S.D., ** $p \leq 0.01$, *** $p \leq 0.001$. The full length of western blotting images was showed in the **Supplementary Fig. 34**.

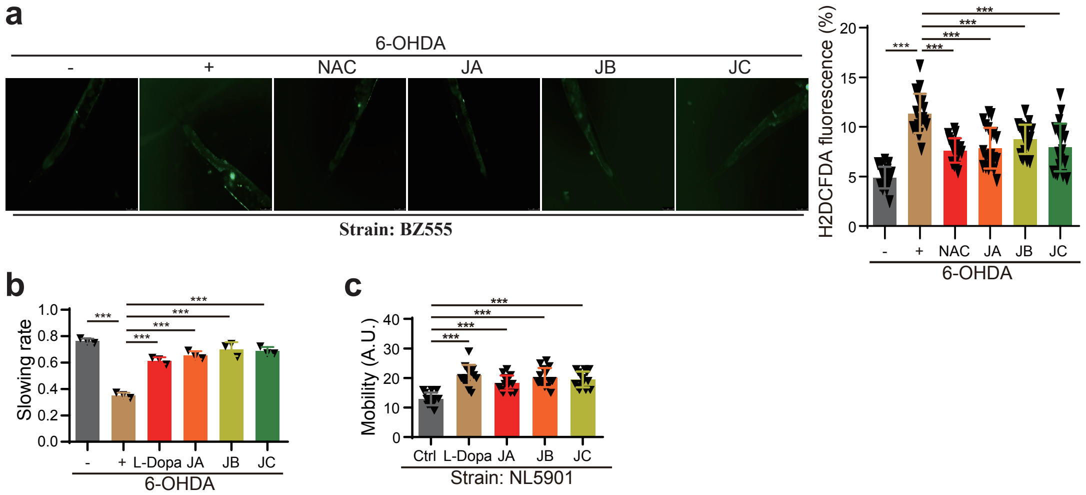

Supplementary Fig. 20 JA, JB and JC possess anti-oxidative and behavioral improvement effects in BZ555 and NL5901 strains. a 6-OHDA-induced BZ555 *C. elegans* were treated with JA (100 μM), JB (100 μM), JC (100 μM) or NAC (5 mM) for 72 h. After treatments, the animals were incubated with H_2DCF-DA probe for 30 min, and the representative images of worms were captured by a fluorescence microscope (magnification, ×20, Scale bar: 100 μm). Bar chart indicated the relative H_2DCF-DA fluorescence intensity. Bars, S.D., *** $p \leq 0.001$, n=20. **b** 6-OHDA-induced BZ555 *C. elegans* were treated with JA (100 μM), JB (100 μM), JC (100 μM) or L-Dopa (2 mM) for 72 h. After treatments, the food-sensing was assayed by counting the body bends of worms for 20s in the presence or absence of food. Bar chat indicated the slowing rates. Bars, S.D., *** $p \leq 0.001$, n=20. **c** NL5901 *C. elegans* were treated with JA (100 μM), JB (100 μM), JC (100 μM) or L-Dopa (2 mM) for 72 h. After treatments, the number of body bends was counted in 20s. Bar chart indicated the mobility of worms in arbitrary units (A.U.).

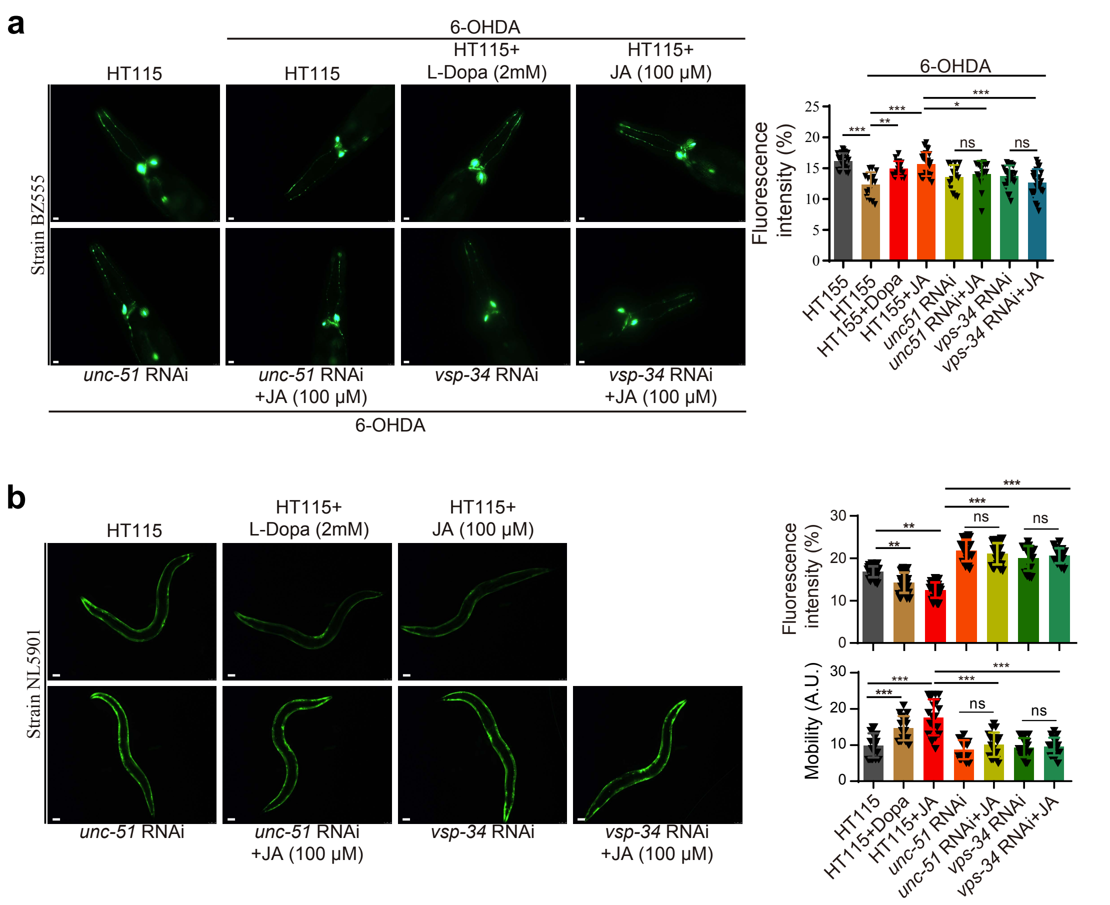

Supplementary Fig. 21 JA exerts neuroprotective effects in BZ555 and NL5901 *C. elegans* via the autophagy-related genes *unc-51* and *vps-34*. a 6-OHDA-induced BZ555 *C. elegans* were treated with JA (100 μM), L-Dopa (2 mM) or JA (100 μM) in the presence or absence of *unc-51* RNAi or *vps-34* RNAi bacteria to RNAi knockdown *unc-51* or *vps-34* gene in worms. After 72 h treatment, the representative images of worms were captured by a fluorescence microscope (magnification, ×40, Scale bar: 10 μm). Bar chart indicated the relative fluorescence intensity. Bars, S.D., ns, no significance, * $p \leq 0.05$, *** $p \leq 0.001$, n=20. **b** NL5901 *C. elegans* were treated with JA (100 μM), L-Dopa (2 mM) or JA (100 μM) in the presence or absence of *unc-51* RNAi or *vps-34* RNAi bacteria to RNAi knockdown *unc-51* or *vps-34* gene in worms. After 4 days treatment, the representative images of worms were captured by a fluorescence microscope (magnification, ×10, Scale bar: 50 μm). Bar chart indicated the relative fluorescence intensity. Bars, S.D., ns, no significance, ** $p \leq 0.01$, *** $p \leq 0.001$, n = 20. At the same time, the number of body bends was counted in 20s. Bar chart indicated the mobility of worms in arbitrary units (A.U.). Bars, S.D., ns, no significance, *** $p \leq 0.001$, n = 20

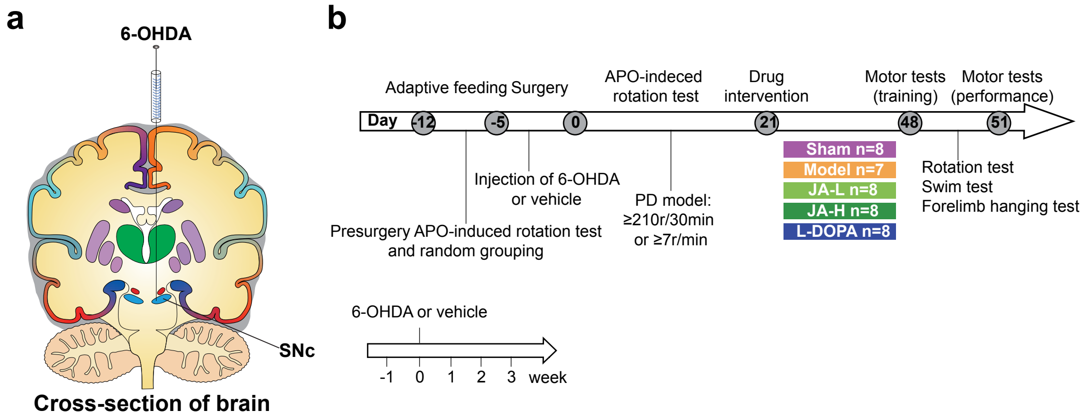

Supplementary Fig. 22 Design of the animal experiment in 6-OHDA-induced rats. a Sketch of a unilateral injection of 6-OHDA into the right midbrain substantia nigra pars compacta (SNc) the nigrostriatal tract in rats. **b** Timeline of the study: animals were subjected to adaptive feeding for 1 week before surgery. The rats showed less than 210 rotations per 30 min in pre-surgery rotational test that were used for experiment. After injection of 6-OHDA or saline, APO-induced rotational test was used to screen the PD model rats. On day 21, rats were administrated with saline, JA (5 mg/kg), JA (10 mg/kg), or L-Dopa (10 mg/kg). The behavioral training including rotational analysis, swimming test, and forelimb hanging test were conducted. Finally, the motor performance tests of rats were performed before decapitation. Rotational analysis was performed on days 48-51. Finally, all animals were sacrificed on day 51 after behavioral test.

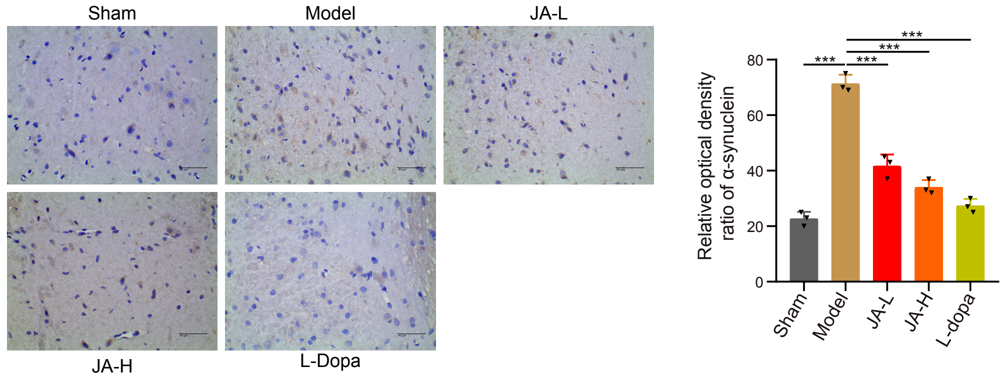

Supplementary Fig. 23 Immunohistochemistry detection of α-synuclein in the corpus striatum of 6-OHDA-induced rats treated with L-Dopa or JA. The representative images were captured by a microscope (magnification, ×40). Scale bar: 40 μm. Bar chart indicates the relative optical density of α-synuclein. Bars, S.D., *** $p \leq 0.001$.

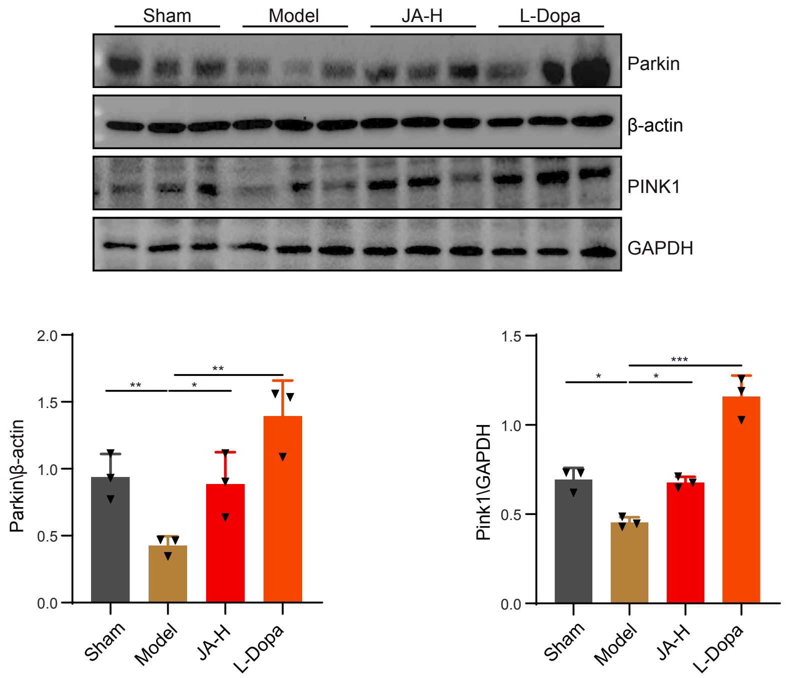

Supplementary Fig. 24 JA activates the Parkin/PINK1 signaling pathway in 6-OHDA-induced rats. Protein expression of Parkin and PINK in brain corpus striatum of 6-OHDA rats treated with L-Dopa or JA. Bar chart indicated the relative expression of the Parkin to β-actin or PINK1/GAPDH. Bars, S.D., * $p \leq 0.05$, ** $p \leq 0.01$. The full length of western blotting images was showed in the **Supplementary Fig. 36**.

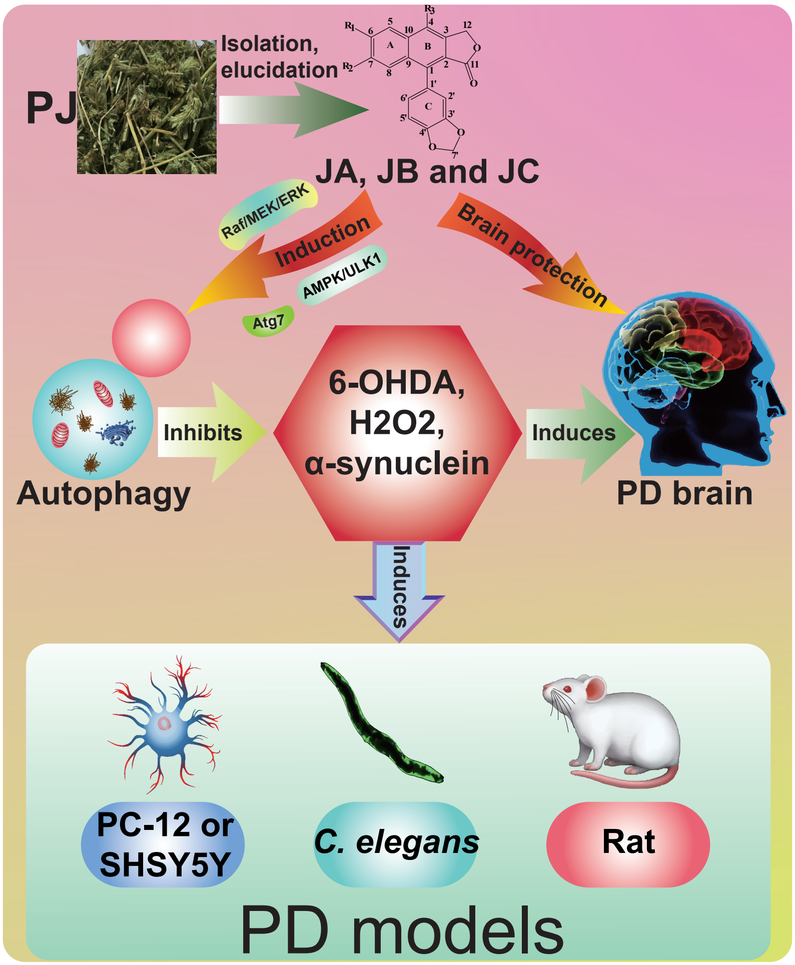

Supplementary Fig. 25 A schematic diagram of the study on PJ. The liganans including JA, JB and JC isolated from PJ exert neuroprotective effect in PD models of 6-OHDA- or H_2O_2-induced cells, or α-synuclein expressed cells, *C. elegans*, and 6-OHDA-induced rats via Atg7, and AMPK dependent but mTOR independent induction of autophagy.

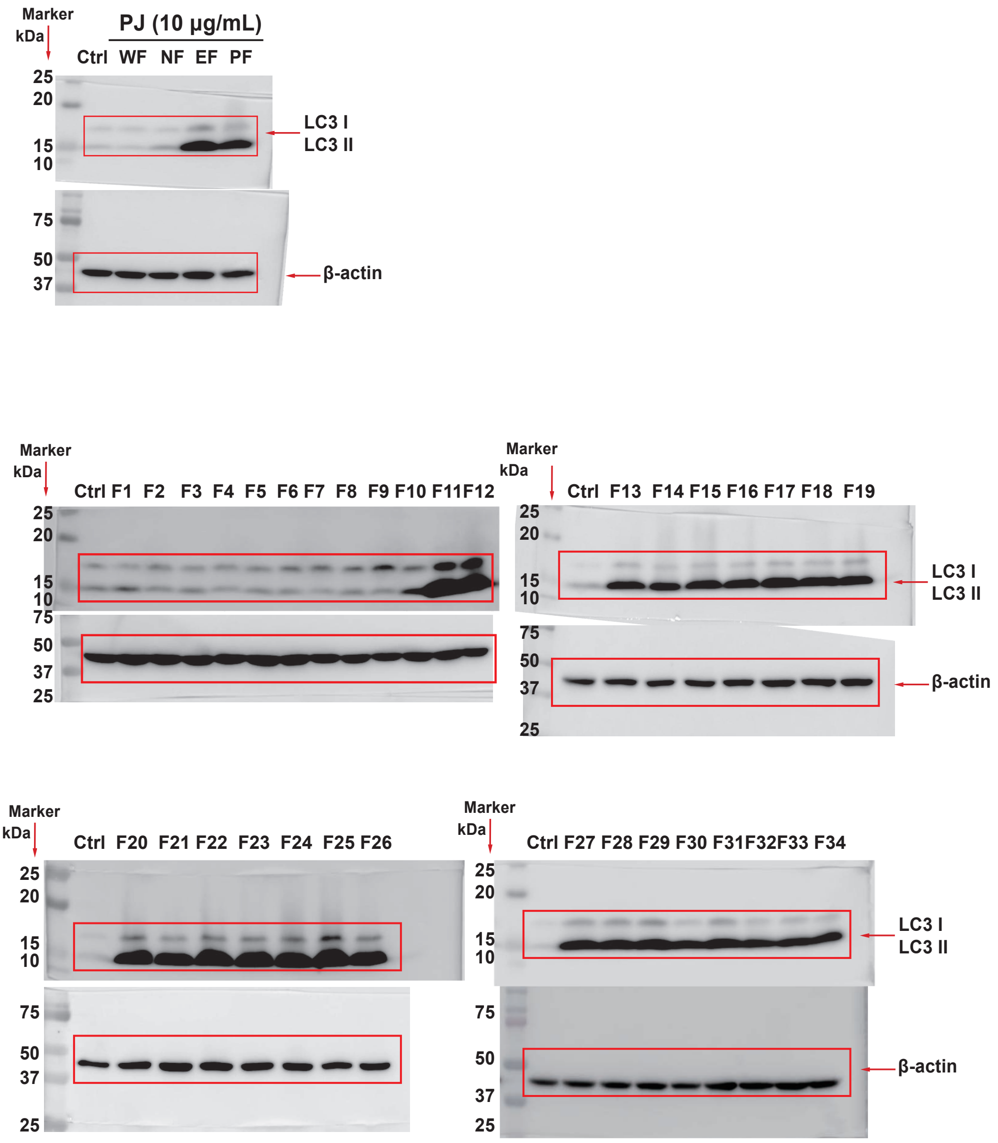

Supplementary Fig. 26 The full-length Western blotting images for **Supplementary Fig. 2e** and **3c**.

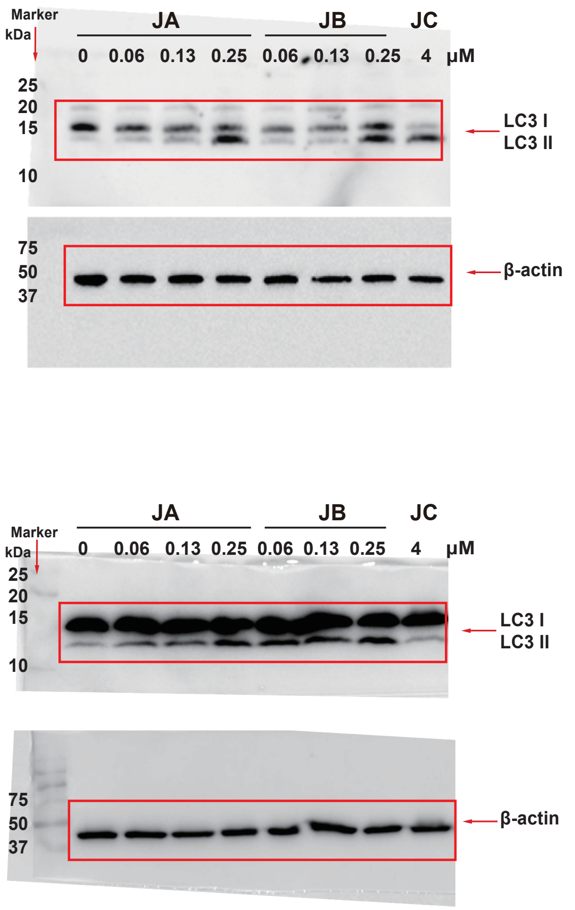

Supplementary Fig. 27 The full-length Western blotting images for **Fig. 1c**.

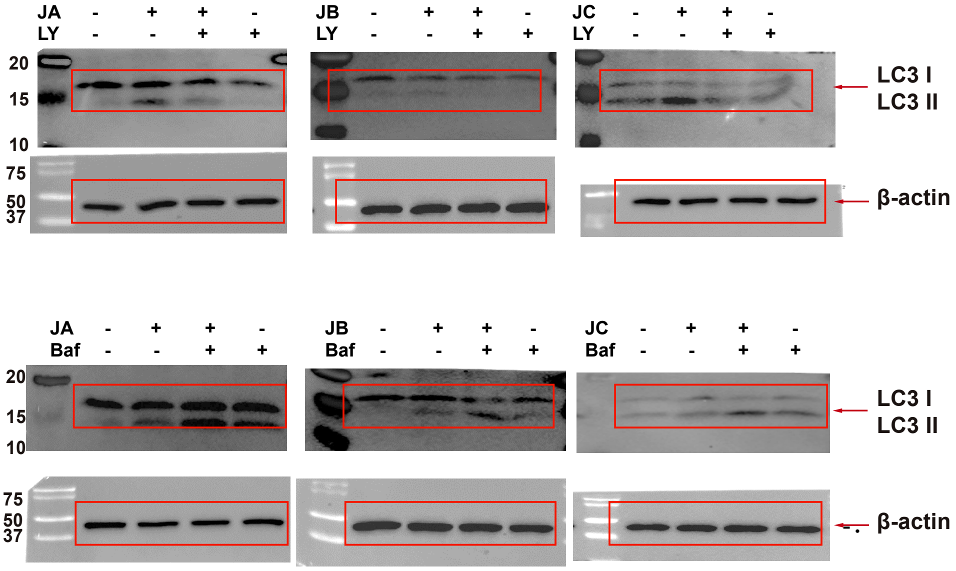

Supplementary Fig. 28 The full-length Western blotting images for **Supplementary Fig. 8b, c**.

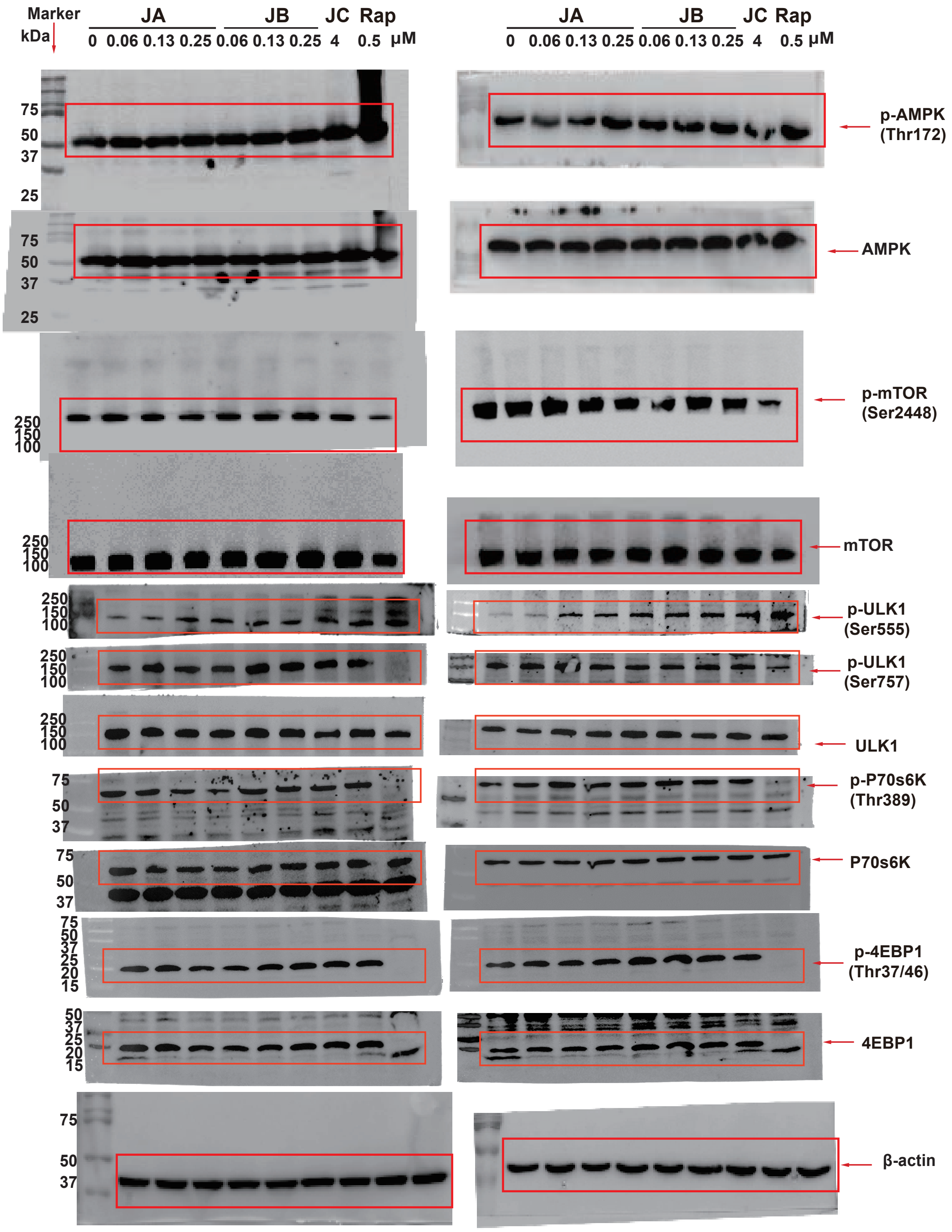

Supplementary Fig. 29 The full-length Western blotting images for **Fig. 1d**.

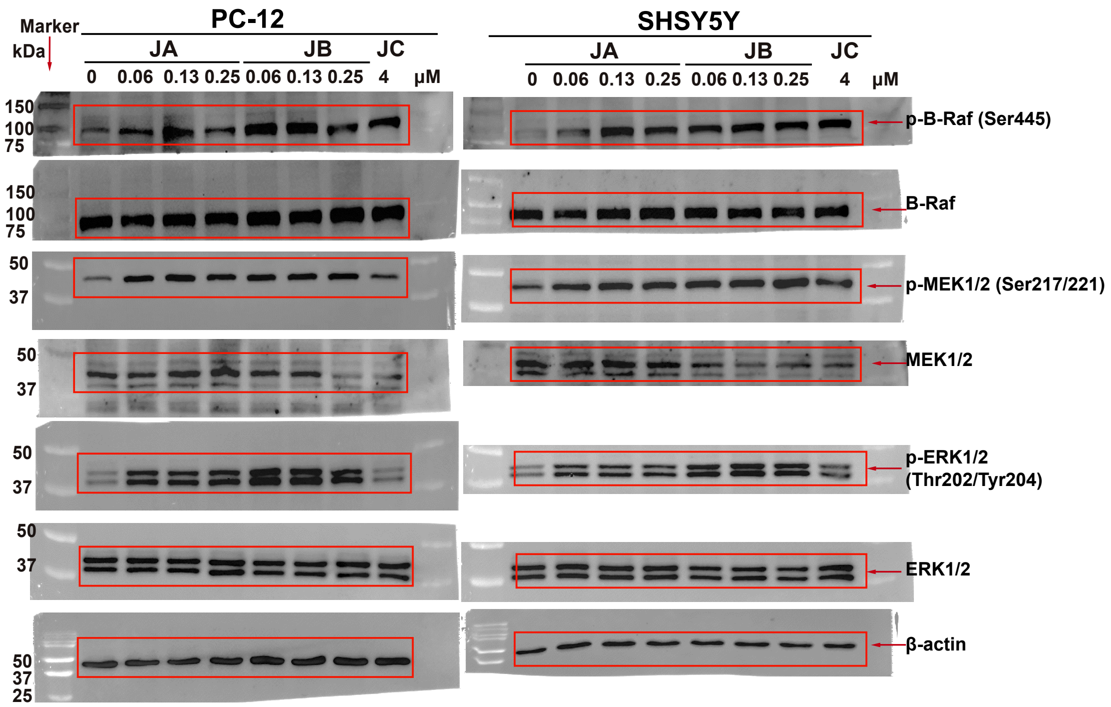

Supplementary Fig. 30 The full-length Western blotting images for **Supplementary Fig. 10**.

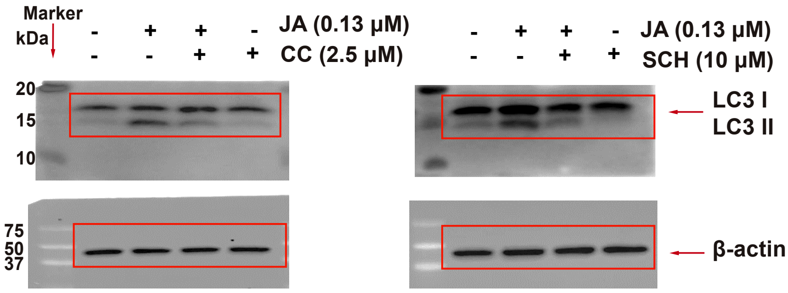

Supplementary Fig. 31 The full-length Western blotting images for **Supplementary Fig. 11**.

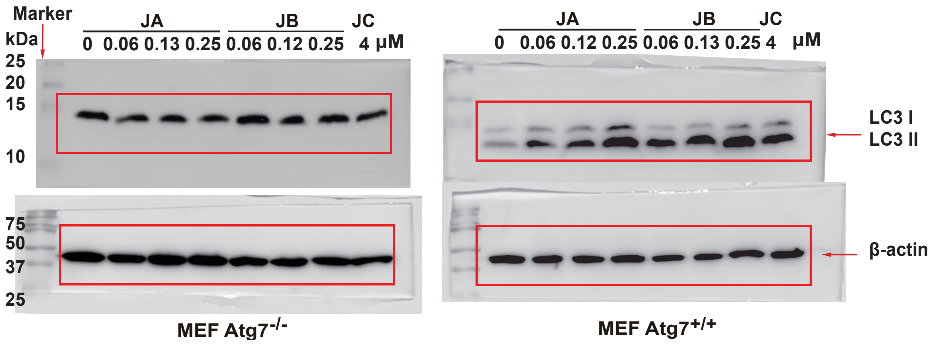

Supplementary Fig. 32 The full-length Western blotting images for **Fig. 1f**.

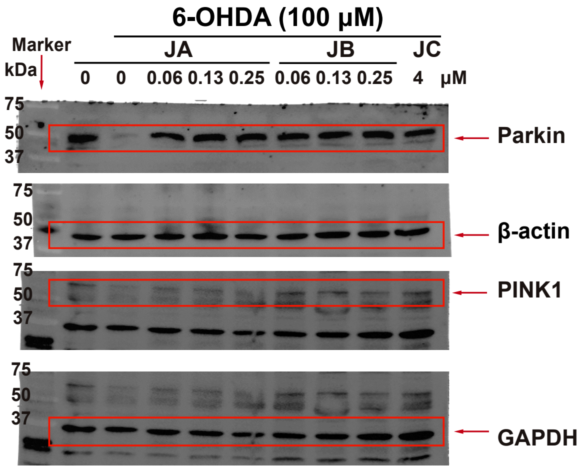

Supplementary Fig. 33 The full-length Western blotting images for **Supplementary Fig. 15**.

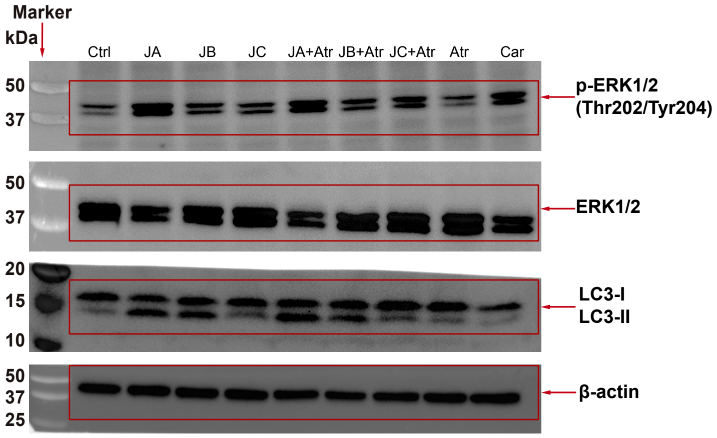

Supplementary Fig. 34 The full-length Western blotting images for **Supplementary Fig. 19**.

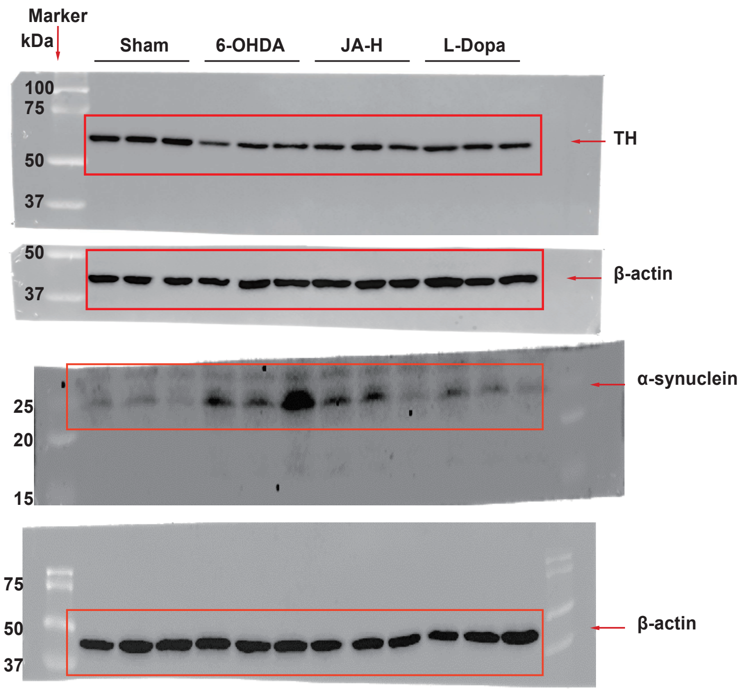

Supplementary Fig. 35 The full-length Western blotting images for **Fig. 1l**.

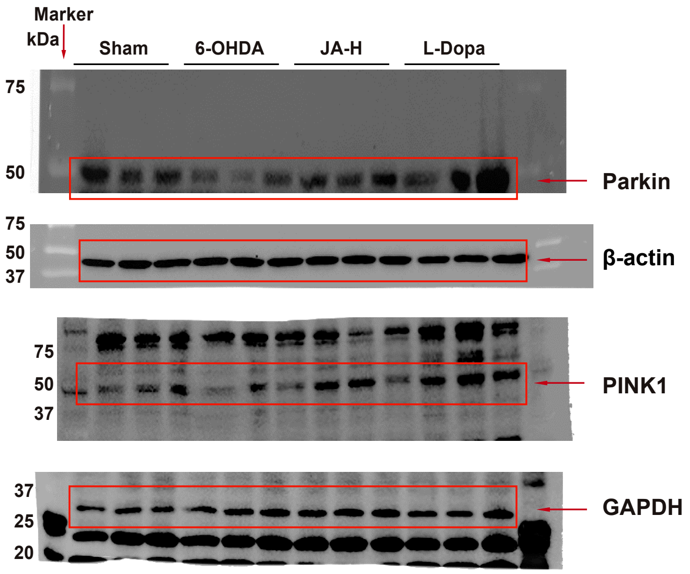

Supplementary Fig. 36 The full-length Western blotting images for **Supplementary Fig. 24**.
